# A two-pronged strategy eliminates dissociation artifacts for high-fidelity neuroimmune single-cell transcriptomics

**DOI:** 10.1038/s42003-026-10609-x

**Published:** 2026-07-06

**Authors:** Yuan Yan, Bo Tang, Pu Liu, Dongmei You, Yi Lv, Jinmeng Yi, Yuzhang Wu, Yiguo Qiu

**Affiliations:** 1 School of Basic Medical Sciences, Southern Medical University, Guangzhou, China; 2 Chongqing International Institute for Immunology, Chongqing, China; 3 Sichuan-Chongqing Joint Key Laboratory of Traditional Chinese Medicine and Immunology, Chongqing, China; 4 Institute of Immunology, Third Military Medical University (Army Medical University), Chongqing, China

**Keywords:** Neuroimmunology, Computational biology and bioinformatics

## Abstract

Single-cell RNA sequencing (scRNA-seq) is revolutionizing neuroimmune research, yet a critical bottleneck persists: the dissociation process introduces pervasive stress-altered transcription (SAT) that obscures genuine in vivo biology. Here, we present a comprehensive two-pronged strategy to eliminate these artifacts in key neuroimmune niches. First, we establish a benchmark by applying an optimized experimental protocol that minimizes dissociation stress. Comparative analysis against conventional methods allows us to map the landscape of SAT artifacts across cell types in the brain and skull bone marrow (SBM), defining distinct, tissue-specific signatures. We then employ random forest-based approach to refine these signatures into powerful SAT gene panels, creating a computational tool for the sensitive detection and removal of artifactual signals. Our experimental approach achieves a ~ 95% reduction in these artifacts, while computational approach alone also has a 60-64% reduction that enables the retrospective correction of published data. This work provides a robust framework to ensure single-cell transcriptomics faithfully captures biological reality, empowering more precise discoveries in neuroimmunology.

## Abstract (web summary)

An experimental and computational framework to minimize dissociation-induced transcriptional artifacts in single-cell RNA sequencing of neuroimmune tissues by defining tissue-specific stress signatures and enabling in silico correction.

## Introduction

Single-cell RNA sequencing (scRNA-seq) is revolutionizing our understanding of biological complexity, enabling unprecedented insights into cellular heterogeneity and transcriptional dynamics across diverse biological systems and tissues. This technology has been particularly transformative in neuroimmunology, illuminating intricate neuro-immune interactions and molecular mechanisms underlying neurological disorders, thereby guiding the discovery of potential therapeutic targets^1–3^. However, the biological fidelity of these powerful insights hinges on a critical prerequisite: preserving authentic in vivo transcriptional states during sample preparation.

The enzymatic dissociation protocols commonly used to isolate cells from the central nervous system (CNS) and its associated immune niches introduce significant stress, profoundly altering cellular gene expression^4^. These alterations, often characterized as dissociation-induced artifacts, which we refer to as stress-altered transcription (SAT), manifest as spurious inflammatory and stress-response signals. Such artifacts can obscure genuine cell states, mask disease-specific molecular signatures, and lead to misinterpretations of cellular communication and function in neuroimmune research^4–6^.

While single-nucleus RNA sequencing (snRNA-seq) offers an alternative by avoiding enzymatic dissociation, it fails to adequately capture mature cytoplasmic mRNAs due to its nuclear transcript bias. This is a significant drawback for decoding authentic cellular states, particularly in immune cells^7^. Consequently, scRNA-seq remains indispensable, especially for animal studies demanding a complete cellular transcriptome, highlighting the critical need to address its inherent dissociation artifacts.

Despite the increasing recognition of these artifacts, a systematic understanding of their impact and effective solutions remains limited, particularly within key neuroimmune niches. Existing studies have often focused on specific brain cell types or regions^4^, thus lacking a comprehensive characterization across the brain’s full cellular landscape. Moreover, the skull bone marrow (SBM) has recently emerged as a crucial interface between systemic and brain immunity^8–11^, yet its effective dissociation for single-cell analysis requires enzymatic digestion, rendering it susceptible to artifacts that remain completely uncharacterized. This knowledge gap leaves a vital neuroimmune niche vulnerable to technical misinterpretation and hinders high-fidelity analysis.

To address these critical limitations and provide a unified solution, we developed a comprehensive, two-pronged strategy. First, we established an optimized experimental protocol that was originally adopted from Marsh et al^4^., which effectively suppresses SAT during tissue dissociation, creating a high-fidelity benchmark for mouse brain and SBM. Second, using this benchmark, we systematically mapped the landscape of dissociation artifacts, defining tissue-specific SAT signatures. We then leveraged these signatures to create a random forest-based computational tool for the sensitive detection and removal of artifactual signals, enabling retrospective correction of existing datasets. This integrated framework could promote the data fidelity in single-cell transcriptomics, ensuring that scRNA-seq data faithfully captures the authentic in vivo biology of neuroimmune tissues and empowering more precise discoveries in health and disease.

## Results

### Multi-cellular susceptibility to dissociation stress-induced artifacts in mouse brain

**Fig. 1:**
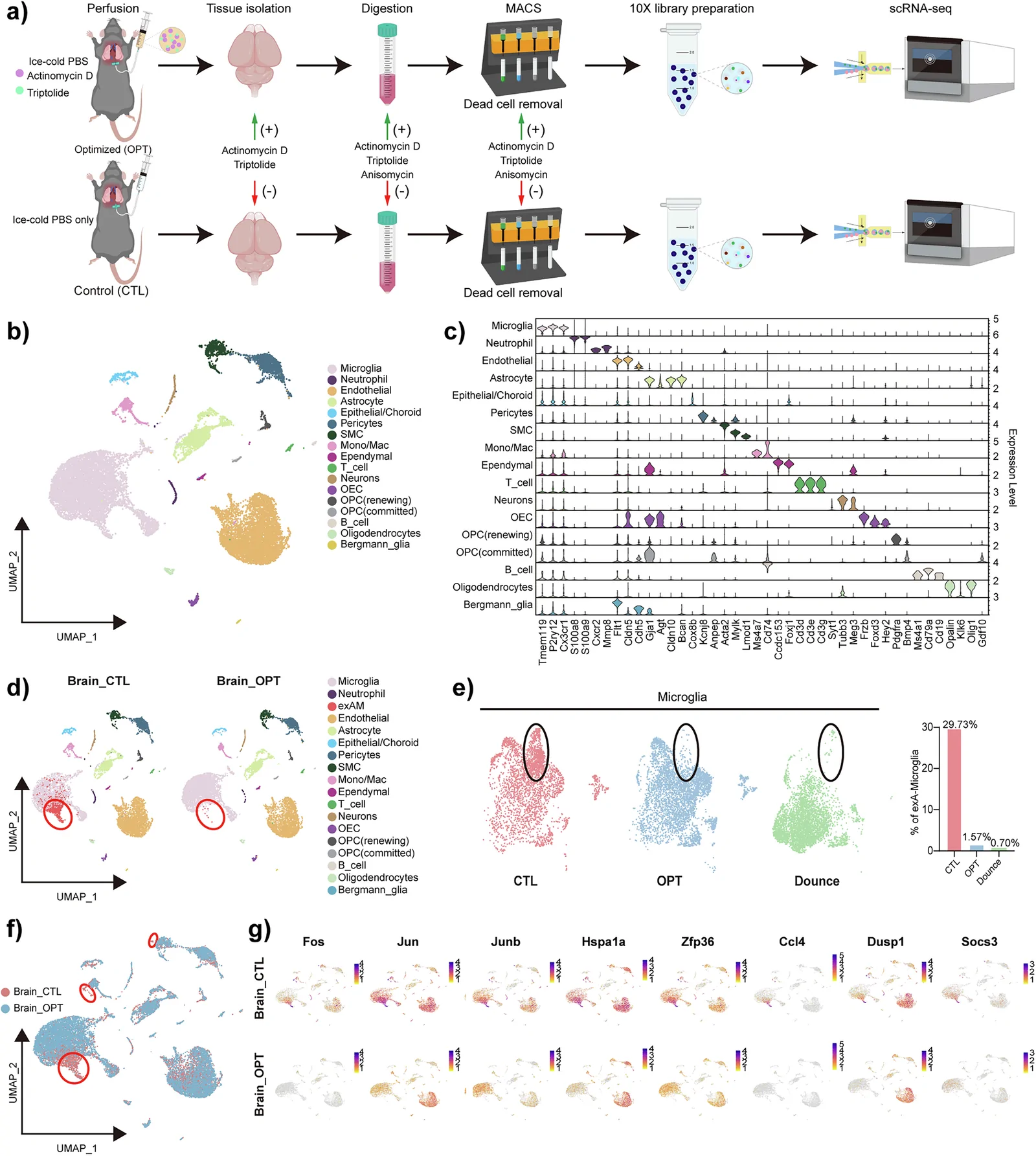
Optimization of single-cell RNA sequencing workflow minimizes artifactual gene expression in mouse brain. **a** Schematic of control (CTL) and optimized (OPT) scRNA-seq workflows for mouse brain. Both protocols use papain-based tissue digestion. The OPT protocol incorporates Actinomycin D and Triptolide during perfusion and tissue isolation, with Anisomycin added during enzymatic digestion to suppress stress-induced transcription. Dead cell removal was performed using MACS separation followed by 10X Genomics library construction and sequencing. All constituent elements of this image were created by the authors. No third-party or previously published materials were used. **b** UMAP visualization of integrated brain cell populations (17 clusters) from CTL and OPT groups (total 18,572 cells from 6 mice; *n* = 3 per group), colored by cell type. SMC: smooth muscle cells; OEC: olfactory ensheathing cell; OPC: oligodendrocyte precursor cell. **c** Violin plots displaying expression of canonical marker genes used for cell type annotation. **d** UMAP plots showing brain cell clusters from CTL (left) and OPT (right) groups separately. A distinct subpopulation of ex vivo activated microglia (exAM, red) is present in the CTL but not the OPT condition. **e** UMAP plot comparing microglia from Brain_CTL, Brain_OPT, and Brain_Dounce (cold mechanical dissociation) samples. exAM populations are highlighted with circles. The bar graph quantifies the percentage of exAM cells within total microglial cells from each condition. **f** Overlay of Brain_CTL (Red) and Brain_OPT (Blue) cells on a UMAP plot. Red circles denote cell regions with differentially elevated expression of exAM signature genes found in Brain_CTL sample. **g** Feature plots showing the expression of the representative exA-genes (e.g., *Fos, Jun, Junb, Hspa1a, Zfp36*, *Ccl4, Dusp1* and *Socs3*) across all brain cell clusters in CTL and OPT groups.

To systematically characterize the susceptibility of diverse brain cell types to dissociation-induced artifacts beyond previously reported microglial signatures^4^, we compared single-cell preparations from mouse brain using a conventional enzymatic dissociation method (CTL) and an optimized protocol (OPT). Our protocol incorporates a transcriptional and translational inhibitor cocktail^4^ with a refined single-cell preparation workflow (Fig. 1a) to minimize stress-induced artifacts^12,13^. Importantly, we verified that this protocol did not compromise RNA quality (Supplementary Fig. 1a) or introduce significant batch effects to the samples, which is essential for downstream analyses (Supplementary Fig. 1b).

After quality control, we successfully analyzed 18,572 cells from six mouse brains and identified 17 transcriptionally distinct clusters annotated using canonical markers (Fig. 1b, c). As anticipated, the microglial cluster from the CTL sample displayed prominent artifactual subclustering, consistent with the previously described ex vivo activated microglia (exAM) signature^4^. In contrast, this effect was substantially reduced in the OPT sample (Fig. 1d).

To confirm that these transcriptional changes specifically resulted from enzymatic dissociation stress, we compared our data to a published dataset generated using cold, mechanical Dounce homogenization (GSM4605687)^4^. The CTL samples showed strong enrichment of the exAM signature, whereas the Dounce samples displayed minimal levels. Strikingly, the OPT method reduced the proportion of exAM signature-positive microglia by 94.7% (from 29.73% to 1.57%), bringing it to levels comparable to the cold dissociation baseline (Fig. 1e). Visualization of representative exAM genes (e.g., *Fos, Dusp1, Hspa1a, Zfp36*) in microglial uniform manifold approximation and projection (UMAP) plot further confirmed the suppression of stress-associated expression by the OPT protocol (Supplementary Fig. 2).

It has been reported that elevated temperature is a major contributor to dissociation stress^14^. To evaluate the OPT approach, we therefore conducted a simplified proof-of-concept experiment using an ex vivo heat shock model in BV2 microglial cells^15^. Both bulk RNA-seq and RT-qPCR analyses confirmed that heat shock (42 °C, 2 hours) could rapidly induce widespread gene expression changes, many of which overlapped with our SAT signature. In particular, heat shock induced a clear upregulation of exAM signature genes, which was effectively mitigated by the inhibitor cocktail (Supplementary Fig. 3 a, b). Further analysis using Gene Set Enrichment Analysis (GSEA) identified significant pathway alterations in heat-stressed BV2 cells, encompassing both canonical stress responses (annotated in blue) and immune-related processes (annotated in red) (Supplementary Fig. 3c, Supplementary Data 1). Beyond microglia, integrated UMAP visualization revealed protocol-dependent clustering separations in monocytes/macrophages (mono/mac) and smooth muscle cells (SMCs), mirroring the observation in microglia (Fig. 1f). Consistent with these clustering patterns, enrichment of representative exAM signatures was most pronounced in these three cell types (Fig. 1g). Together, these findings establish enzymatic dissociation artifacts as a multi-lineage phenomenon^13,16,17^.

### Identification of a conserved stress-altered transcriptional (SAT) signature across brain cell types

**Fig. 2:**
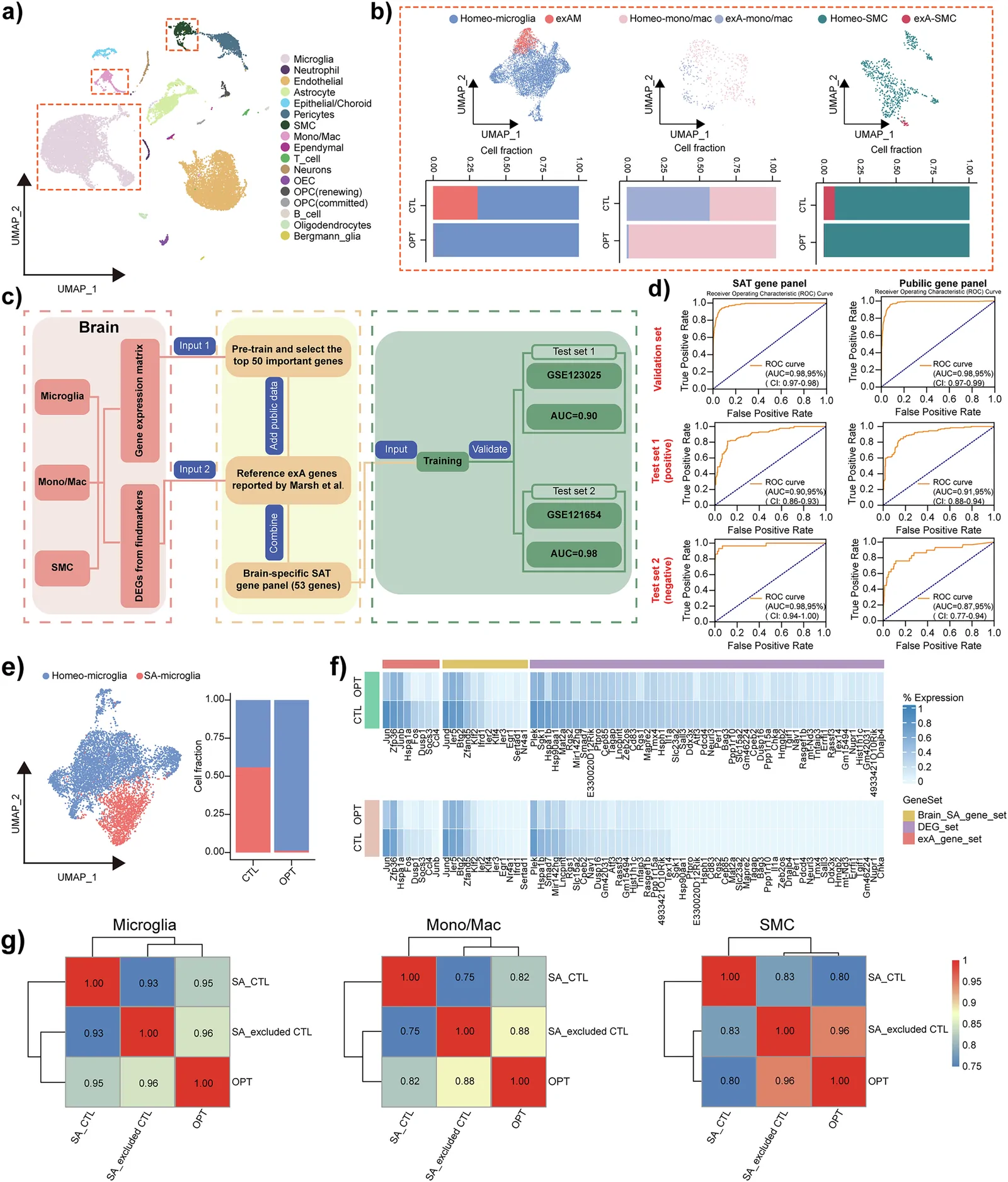
Single-cell transcriptomic profiling identifies stress-altered transcription (SAT) signatures across multiple brain cell types. **a** UMAP visualization of brain cell populations from scRNA-seq analysis. Red boxes indicate cell types exhibiting high sensitivity to enzymatic dissociation stress. **b** Expression of ex vivo activated (exA) gene signatures in microglia (left), monocytes/macrophages (middle), and smooth muscle cells (SMCs, right), showing distribution of exA-gene-expressing cells and homeostatic counterparts. Corresponding bar graphs quantify the proportion of cells expressing exA-gene signatures within each cell type. **c** Computational workflow for identifying brain-specific SAT signatures. Red box: Transcriptomic profiles from stress-sensitive cell types (microglia, Mono/mac, SMC) were integrated and subjected to two parallel approaches: random forest classification (Input 1) and differential expression analysis (FindMarkers, Input 2). Yellow box: Key genes from Input 1 and Input 2 were combined with established exAM markers from Marsh et al. to generate the brain-specific SAT gene panel. Green box: This panel was then used to train a classifier for discriminating stress-altered (SA) cells in positive (test set 1, ex vivo activated) and negative (test set 2, ex vivo inactivated) test sets. **d** Receiver operating characteristic (ROC) curves evaluating the performance of SAT and exAM signature models. Models were trained in our dataset (training set GSE294852) and tested on independent datasets: negative testing set (GSE121654, containing ex vivo inactivated microglia) and positive testing set (GSE123025, containing identified ex vivo activated microglia). **e** UMAP plots showing distribution of stress-altered (SA)-gene expressing cells and homeostatic counterparts in the microglial population. Corresponding bar graphs show the percentage of cells expressing SAT signatures. **f** Heatmap visualization of the top 50 differentially expressed genes (DEGs) between CTL and OPT conditions in microglia, comparing uncorrected data (labeled in green) with SA-corrected data (labeled in pink, after in silico removal of SAT-expressing cells). Color bars indicate expression levels and gene set categories. **g** Spearman correlation analysis of transcriptomic profiles after computational correction. Microglia (left), monocytes/macrophages (middle), and SMCs (right) from CTL samples were subdivided into SA-positive (SA_CTL) and SA-excluded populations, then compared with OPT samples using complete transcriptome profiles.

Given the multi-lineage susceptibility to dissociation artifacts, we aimed to define a conserved stress-altered transcriptional (SAT) signature that could capture this shared stress response. We focused on microglia, monocytes/macrophages and SMCs, which exhibited the most pronounced artifact-related subclustering (Fig. 2a). In the CTL sample, cells from these populations were classified as ex vivo activated (exA-cells) or homeostatic (Homeo-cells) based on established exAM gene signatures, whereas this separation was largely abolished in the OPT sample (Fig. 2b).

To establish a robust and generalizable SAT signature, we developed an integrative bioinformatics and machine learning framework. This approach systematically combines differential expression analysis with supervised machine learning models to pinpoint a minimal set of candidate genes with high predictive power (Fig. 2c). Briefly, we combined transcriptomic profiles from all three sensitive cell types and trained a random forest model to rank genes by their importance in discriminating exA- versus Homeo-cells across all cell types (Supplementary Data 2). A model performance test was conducted to choose the feature threshold for downstream analysis (Supplementary Fig. 4), and the Top 50 genes were chosen as gene_set 1. Parallel differential expression analysis (FindMarkers, *p* < 0.05, logFC > 0.5) yielded gene_set 2. The intersection of these sets, supplemented with key exAM genes, produced a candidate set of 53 SAT-associated genes (gene_set 3, Supplementary Data 3). It is worth noted that gene_set1 contains the majority of genes that form our 53 SAT signature, highlighting the necessity of using the random forest model for SAT gene screening. Specifically, gene_set 1 and gene_set 2 share approximately 12% of gene_set 1 genes but 54.5% of gene_set 2 genes, and gene_set 1 shares 14% of its genes with the previously established exAM marker set (Supplementary Fig. 5a, Supplementary Data 4). Moreover, gene_set1 includes 37 unique genes that are not captured by gene_set2 or the established exAM marker set. Those unique genes are important for precise discrimination of stress-altered (SA) cells. They not only include genes involved in stimulus response (e.g., *Egr1, Atf3, Fosb, Nfkbiz, Ppp1r15a*), cytokine/immune response (e.g., *Ccl3, Il10ra, Cd83, Mafb, Siglech* and *Runx1*), intracellular signal transduction (e.g., *Rgs1/2, Rasgef1b, Rhob, Pag1* and *Plek*) and stress response (e.g., *Hspa1b, Hsp90ab1, Txnip* and *Ppp1r15a*), but also feature genes that showed high importance in the random forest classifier and whose co-expression patterns effectively distinguished exA cells from homeostatic counterparts. These genes are enriched in pathways such as“regulation of transcription from RNA polymerase II promoter in response to stress”,“regulation of ENA-templated transcription in response to stress”, “regulation of G protein-coupled receptor signaling pathway”and“positive regulation of phosphate activity” (Supplementary Fig. 5b) and other immune-related pathways (Supplementary Data 5).

This 53-gene signature exhibited high accuracy in discriminating cell states in both internal and external datasets, outperforming previously reported exAM signatures in public benchmarks^4,18^ by accurately identifying artifactually activated (90% accuracy) and inactivated cells (98% accuracy) (Fig. 2d). To evaluate model performance at the biological replicate level, we used another external dataset (GSE152182)^4^. Our model achieved consistent performance across individual animals, with area under curve (AUC) values of 0.90 (Fig. 2d) and 0.92 (Supplementary Fig. 6). From this set, we identified a panel of representative genes (e.g., *Jun, Zfp36, Jund, Ier5, Btg2, Junb, Fos, Klf2, Zfand5, Ccl4, Dusp1, Socs3, Ifrd1, Egr1, Ier2, Ier3, Sertad1, Klf4 and Nr4a1*) that are consistent upregulated across all three cell types (Supplementary Fig. 7a). Feature plots further confirmed their broad expression across brain cell populations (Supplementary Fig. 7b).

We validated the sensitivity of the newly discovered SAT signature by re-clustering cells based on these 53 genes, which effectively partitioned microglia, monocytes/macrophages, and SMCs into distinct homeostatic cells (Homeo-cells) and stress-altered cells (SA-cells) (Fig. 2e, Supplementary Fig. 8a). The SAT-based classification identified a larger proportion of affected cells in the CTL sample (e.g., ~2-fold increase in microglia) compared to the exAM-based approach, with minimal changes in OPT samples, confirming that our SAT signature are a robust and comprehensive probe for systematically identifying and quantifying dissociation-induced transcriptional changes in single-cell data.

Compared to Dounce-isolated microglia, CTL microglia showed an artifactual activation profile, evidenced by the upregulation of 70 genes encompassing both exA-genes and SAT genes (Supplementary Fig. 8b, Top 50 shown). We next evaluated the utility of our SAT signature for in silico correction of scRNA-seq data. Initial differential expression analysis between uncorrected CTL and OPT microglia identified 82 genes upregulated in CTL samples. Strikingly, 69 of these genes overlapped with the 70 genes upregulated in CTL versus Dounce-isolated microglia, strongly supporting that OPT samples more closely reflect the in vivo state (Fig. 2f, Supplementary Fig. 8b).

To assess the effectiveness of in silico artifact removal, we performed DEGs analysis between CTL and OPT microglia after computationally eliminating cells expressing our SAT signature (SA-cells). In uncorrected data, differential expression was observed across exAM genes (exA_gene_set), SAT genes (Brain_SA_gene_set), and other genes (DEG_set, top 50 shown). However, removal of SAT-expressing cells dramatically reduced the number of stress-induced differentially expressed genes by 60% (to 20 genes) in the remaining cells (Fig. 2f). Similar correction efficacy was observed in monocytes/macrophages and SMC (Supplementary Fig. 8c, top 50 shown).

Spearman correlation analysis further validated the correction efficacy, showing that SA-excluded CTL microglia closely resembled OPT microglia (coefficient = 0.96, *p* = 3.62 × 10^-25^), indicating effective normalization toward an in vivo-like state (Fig. 2g). This correction strategy demonstrated consistent effectiveness across other susceptible brain cell types. In monocytes/macrophages, the correlation between CTL and OPT populations increased from 0.82 to 0.88 (7.3% improvement, p = 8.72×10^-27^) following SA-cell removal. Even more pronounced enhancement was observed in SMCs, where the correlation coefficient rose from 0.80 to 0.96 (20% improvement, *p* = 2.89 × 10^−^^11^), suggesting the broad utility of our SAT signature in purifying homeostatic cell populations (Fig. 2g).

To evaluate whether the correction altered global cellular taxonomy, we employed the Adjusted Rand Index (ARI), which quantifies similarity between clusterings. A key strength of the ARI is its ability to distinguish between refinements in cell distribution within clusters and fundamental alterations to the clustering landscape^19–21^. We found that SA-cell removal significantly improved clustering stability, with the ARI score increasing by 8.82% (Supplementary Fig. 8d). This result confirms that our correction enhanced data fidelity by purifying cell populations without distorting the underlying biological structure. Collectively, these results establish that computational exclusion of stress-altered cells based on the SAT signature effectively enriches for transcriptomes that faithfully reflect in vivo biology and substantially improves the reliability of downstream analyses.

### Enzymatic digestion generates stress-induced artifacts in multiple cell types of skull bone marrow (SBM)

**Fig. 3:**
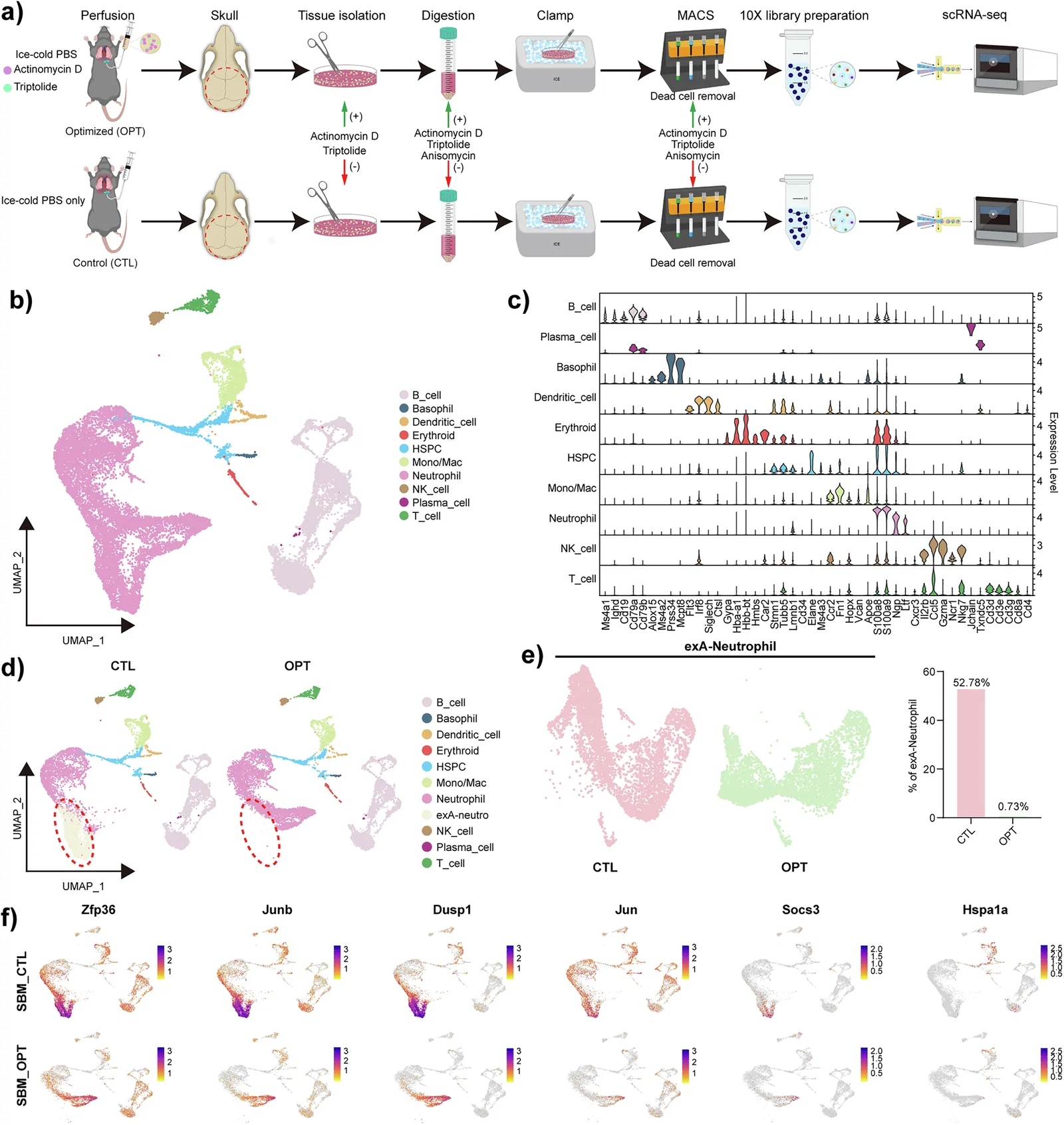
Optimization of scRNA-seq workflow minimizes artifactual gene expression in mouse skull bone marrow (SBM). **a** Schematic of CTL and OPT scRNA-seq workflows for mouse SBM. The OPT protocol incorporates Actinomycin D and Triptolide during perfusion and tissue isolation, with Anisomycin added during enzymatic digestion to suppress stress-induced transcription. Dead cells were removed by MACS separation prior to 10X Genomics library preparation. All constituent elements of this image were created by the authors. No third-party or previously published materials were used. **b** UMAP visualization of integrated SBM cell clusters (10 clusters) from CTL and OPT samples (total 20,470 cells from 6 mice; *n* = 3 per group), colored by cell type. HSPC: Hematopoietic stem and progenitor cell; NK cell: natural killer cell. **c** Violin plots showing expression of canonical marker genes used for SBM cell type identification. **d** UMAP plots showing SBM cell clusters from CTL and OPT groups separately, demonstrating a distinct neutrophil subpopulation (circled) present in CTL but diminished in OPT samples. **e** UMAP plots comparing neutrophil populations from SBM_CTL and SBM_OPT conditions. exA-gene signature-expressing subpopulations are circled. Bar graph quantifies the proportion of exA-neutrophils in each condition. **f** Feature plots showing the expression of the representative exA-genes (e.g., *Dusp1, Junb, Jun, Socs3, Hspa1a* and *Zfp36*) across all SBM cell clusters in SBM_CTL and SBM_OPT groups.

Unlike long bone marrow, efficient isolation of SBM cells for scRNA-seq typically requires enzymatic digestion to ensure adequate cellular yield and diversity^10,22^. With the emerging needs for scRNA-seq studies on SBM in neuroimmunology fields^8,10,23,24^, it is necessary to identify and eliminate dissociation-related artifacts in this niche. We therefore optimized the conventional enzymatic isolation (CTL method) by introducing a post-digestion clamp step to reduce processing time and incorporating an inhibitor cocktail during key steps (OPT method) (Fig. 3a). Again, the inhibitor treatment did not adversely affect cell viability or RNA integrity (Supplementary Fig. 9a), and there were no significant batch effects when integrating the samples for analysis (Supplementary Fig. 9b)

After quality control, we analyzed 20,470 cells from six mice and identified 10 major cell clusters in mouse SBM based on established markers (Fig. 3b, c). Neutrophils exhibited clear subclustering differences between CTL and OPT groups (Fig. 3d), reminiscent of artifact patterns previously observed in microglia. The exA-cell signature genes were highly enriched in a distinct neutrophil subcluster in the CTL sample (Supplementary Fig. 10), and this artifact was markedly suppressed by the OPT method, which reduced the proportion of exA-neutrophils from 52.78% to 0.73% (Fig. 3e). Although other populations did not show obvious subclustering by UMAP visualization alone (Fig. 3d), potentially indicating its limited ability to distinctively identify dissociation stress-altered cells in SBM. Projecting the exA-cell signature genes onto the UMAP plot of all SBM cell clusters revealed significant enrichment in monocytes/macrophages and hematopoietic stem and progenitor cells (HSPCs) of CTL-SBM in contrast to OPT-SBM (Fig. 3f). This suggests these cell types are also susceptible to dissociation stress.

**Fig. 4:**
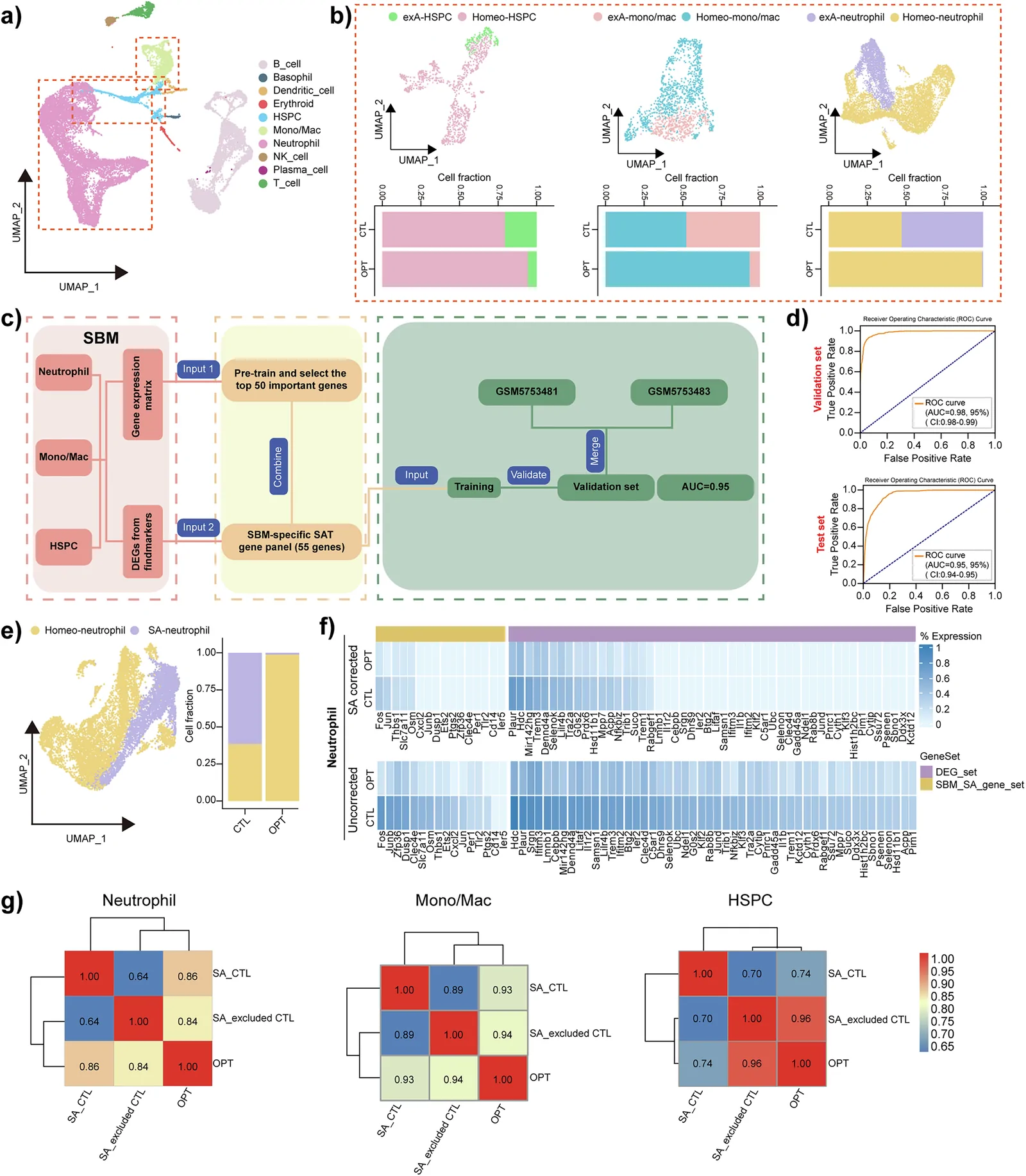
Identification and validation of SAT signatures in SBM. **a** UMAP visualization of SBM cell populations from scRNA-seq analysis. Red boxes indicate cell types exhibiting high sensitivity to enzymatic dissociation stress. **b** Expression patterns of ex vivo activated (exA) gene signatures in HSPCs (left), monocytes/macrophages (middle), and neutrophils (right), showing distribution of exA-gene expressing cells and homeostatic counterparts. Corresponding bar graphs quantify the proportion of cells expressing exA-gene signatures within each cell type. **c** Computational workflow for identifying SBM-specific SAT signatures. Red box: Transcriptomic profiles of stress-sensitive cell types (neutrophil, Mono/mac, HSPC) were integrated and subjected to random forest classification (Input 1) and differential expression analysis (FindMarkers, Input 2) to generate two sets of candidate SAT genes. Yellow box: The SBM-specific SAT gene panel was generated by combining the key genes from Input 1 and Input 2. Green box: This panel was subsequently used to train a classifier for discriminating stress-altered (SA) cells in external validation datasets. **d** Receiver operating characteristic (ROC) curves evaluating SBM-specific SAT signature performance. Models were trained in our dataset (training set, GSE294852), and tested on public datasets: (validation set, GSM5753481 and GSM5753483). **e** UMAP plots showing distribution of SA signature-expressing cells and homeostatic counterparts in the neutrophil population. Corresponding bar graphs show the percentage of cells expressing SAT signatures. **f** Heatmap visualization of top 50 DEGs between CTL and OPT conditions in neutrophils, comparing uncorrected data with SA-corrected data (after in silico removal of SAT-expressing cells). Color bars indicate expression levels and gene set categories. **g** Spearman correlation analysis of transcriptomic profiles after computational correction. Neutrophils (left), monocytes/macrophages (middle), and HSPCs (right) from the CTL sample were subdivided into SA-positive (SA_CTL) and SA-excluded populations, then compared with OPT samples using complete transcriptome profiles.

Indeed, the subclustering of those 3 susceptible cell clusters (Fig. 4a) confirmed that exA-signatures clearly separated neutrophils into exA- and Homeo-cells but performed poorly in HSPCs and monocytes/macrophages (Fig. 4b), indicating limited utility of microglia-derived exA-markers in SBM. Given the potential for tissue-specific differences in response to dissociation stress, we developed a tissue-specific SAT signature for SBM (Fig. 4c). Transcriptomic profiles from neutrophils, HSPCs, and monocytes/macrophages were pooled to train a random forest model; we combined transcriptomic profiles from all three sensitive cell types and trained a random forest model to rank genes by their importance in discriminating exA- versus Homeo-cells across all cell types (Supplementary Data 6). A model performance test was conducted to choose the feature threshold for downstream analysis (Supplementary Fig. 11). Although the optimal performance was observed at top 60 features (0.9451 vs. 0.9338 at top 50), the performance difference between top 50 and top 60 was minimal in practical terms. Overall, model performance was robust to variations in the number of selected features within a broad range. To maintain consistency across tissues and facilitate straightforward application by readers, we adopted the same top 50 threshold for both brain and SBM, so that the Top 50 genes were chosen as gene_set 1. In parallel, differential expression analysis was performed using the FindMarkers() function (*p*-value < 0.05, logfc>0.5) to generate gene_set 2. The overlap of these two gene sets produced a refined candidate list of 55 genes (gene_set 3, Supplementary Data 7). This signature showed excellent classification performance in both internal (AUC = 0.98) and external^10^ (AUC = 0.95) datasets in discriminating the inactivated vs activated cells (featured with dissociation methods) (Fig. 4d). Due to the scarcity of publicly available SBM single-cell datasets that clearly annotate whether samples were generated with or without enzymatic dissociation, only one external dataset^10^ was used for validation here.

Within this 55-gene set, we identified a core panel of 16 representative genes (including *Clec4e, Thbs1, Zfp36, Tlr2, Junb, Fos, Cd14, Jun, Dusp1, Ptgs2, Slc7a11, Osm, Per1, Ier5, Ets2 and Cxcl2*) (Supplementary Fig. 12a, annotated in red). Feature plots visualization confirmed their distinct expression pattern across susceptible SBM cell types (Supplementary Fig. 12b), and SAT-based subclustering cleanly separated all three types into SA- and Homeo-cell populations (Fig. 4e, Supplementary Fig. 13a).

To further validate the impacts of the SAT signature on identifying stress-altered cells, we performed a DEG analysis comparing CTL and OPT neutrophils. In uncorrected data, we observed differential expression of the identified representative 16 SAT signature, as well as other genes (Fig. 4f, top 50 shown). After computationally removing SA-cells, the number of DEGs was substantially reduced by 64% (Fig. 4f), with similar reductions in HSPCs and monocytes/macrophages (Supplementary Fig. 13b).

Moreover, Spearman correlation analysis revealed strong similarity between SA-excluded CTL cells and OPT cells across all three susceptible cell types (neutrophils: 0.64 → 0.84, *p* = 3.78 × 10^−^^218^; monocytes/macrophages: 0.89 → 0.94, *p* = 5.19 × 10^−24^; HSPCs: 0.70 → 0.96, *p* = 3.66 × 10^−55^; Fig. 4g), supporting effective restoration of in vivo-like transcriptional states. The improved clustering stability, as evidenced by increased ARI scores (Supplementary Fig. 13c), further confirms that our correction enhances data quality without distorting the underlying biological structure. Taken together, these results establish that our SBM-specific SAT signature enables accurate identification and computational removal of stress-altered cells, effectively recovering transcriptomes that faithfully represent in vivo biology.

### Key altered pathways in cells undergoing stress-induced stimulation

As aforementioned, dissociation stress introduces substantial confounding effects in differential expression analysis across both brain and SBM tissues (Figs. 2f, 4f, Supplementary Figs. 5c, 13b). To systematically evaluate the potential impact of these artifacts on biological interpretation, we performed GSEA using DEGs identified between SA-cells and Homeo-cells, focusing on canonical pathways from Reactome and Kyoto Encyclopedia of Genes and Genomes (KEGG) databases (normalized enrichment score, NES > 1). Pathways were considered significant with *p* < 0.05 and FDR < 0.25. However, for exploratory purposes, a few pathways that did not meet the FDR threshold are still shown in the figures, with clear annotations and highlighting in Supplementary Data 8 (annotated in red).

**Fig. 5:**
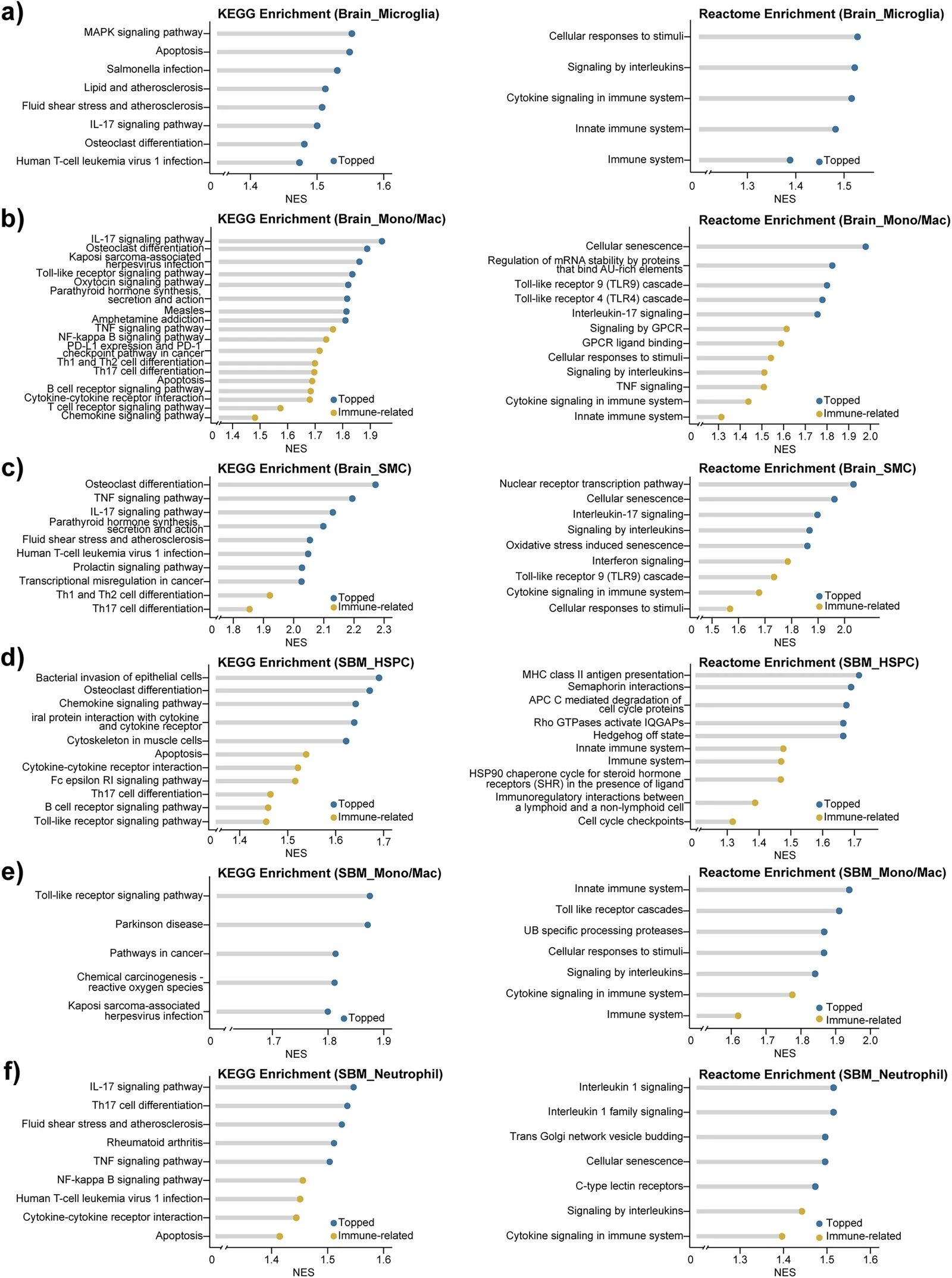
Pathway enrichment analysis reveals immune-related signaling alterations in SAT-associated cells across brain and SBM. **a**–**f** Gene Set Enrichment Analysis (GSEA) of KEGG (left) and Reactome (right) pathways using DEGs from SAT-enriched cell populations in the brain and SBM. Normalized enrichment scores (NES) are shown for selected significantly upregulated pathways. Yellow dots denote immune-related pathways; blue dots indicate the most significantly enriched pathways across all categories. **a** Brain microglia: Enriched KEGG pathways include MAPK signaling, apoptosis, and IL-17 signaling. Reactome analysis highlights enrichment in cytokine signaling and innate immune system pathways. **b** Brain monocytes/macrophages: Both KEGG and Reactome analyses reveal enrichment in IL-17 signaling, Toll-like receptor signaling, TNF signaling, and innate immune system pathways. **c** Brain SMCs: Top KEGG pathways include TNF signaling, osteoclast differentiation, and IL-17 signaling. Reactome enrichment reveals activation of nuclear receptor transcription and cytokine-related signaling. **d** SBM HSPCs: Enriched KEGG pathways show immune-related signaling such as chemokine signaling, cytokine interaction, and Toll-like receptor signaling. Reactome pathways include MHC class I antigen presentation and Hedgehog signaling. **e** SBM monocytes/macrophages: KEGG enrichment identifies pathways including Toll-like receptor signaling and oxidative stress responses. Reactome analysis highlights cytokine signaling and innate immune activation. **f** SBM neutrophils: enriched KEGG pathways include IL-17 and TNF signaling, NF-κB signaling, and apoptosis. Reactome enrichment shows upregulation in interleukin signaling, cellular senescence, and C-type lectin receptor pathways.

We observed that the top dissociation stress-altered pathways for microglia were predominantly associated with cellular responses to stimuli (a hallmark of IEGs activation)^25^, apoptosis (indicative of enzymatic stress)^26^ and immune responses (Fig. 5a). As aforementioned, the simplified experiment in BV2 microglial cells under controlled heat shock conditions confirmed that these pathway alterations may be mainly contributed by temperature stress during dissociation (Supplementary Fig. 3c). Among those 71 temperature-sensitive pathways identified, 14 were immune-related (annotated with red) and 6 were stress-specific (annotated with blue). Similar patterns emerged in other brain cell types. Monocytes/macrophages and SMCs showed significant enrichment in immune response pathways, alongside classical heat stress-stimulated pathway alterations, e.g., cellular senescence and oxidative stress-induced senescence^27,28^ (Fig. 5b). Systematic screening of enriched pathways (NES > 1) revealed that these alterations collectively create a false “activated” phenotype, characterized by elevated inflammatory signaling and a false proinflammatory state in monocytes/macrophages^29,30^. A similar pattern was also observed in brain SA-SMCs (Fig. 5c). Together, these findings indicate that SA-cells in the brain exhibit coordinated activation of both stress response and immune pathways, potentially leading to misinterpretation of cellular states. For Reactome analysis, some pathways in Mono/mac (e.g., TNF signaling and innate immune system) and SMCs (e.g., oxidative stress-induced senescence, interferon signaling, Toll-like receptor 9 cascade, cytokine signaling in the immune system, and cellular responses to stimuli) did not reach FDR significance (Supplementary Data 8, annotated in red). Caution should therefore be exercised when interpreting these pathways for the corresponding cell types.

Parallel analysis in SBM yielded consistent results. Neutrophils, monocytes/macrophages, and HSPCs all showed strong enrichment in dissociation stress-associated pathways, including cellular response to stimuli and cellular senescence, with particularly pronounced activation of immune-related pathways (NES > 1.4, Fig. 5d–f). These alterations may lead to an artifactual proinflammatory signature across multiple SBM cell types. It should also be noted that several pathways in neutrophils did not reach FDR significance, including Reactome pathways (e.g., Trans Golgi network vesicle budding, C-type lectin receptors, and Cytokine signaling in immune system) and KEGG pathways (e.g., Th17 cell differentiation and Rheumatoid arthritis).

### SAT gene signature represents a powerful tool for in silico correction

Our findings establish that enzymatic dissociation induces widespread ex vivo transcriptomic artifacts across cell types, highlighting the critical need for both optimized isolation protocols and computational correction methods. To demonstrate the practical utility of our SAT signatures for retrospective data refinement, we reanalyzed a published scRNA-seq study investigating chlorogenic acid (CHA) effects in the glioma immune microenvironment^31^. The original study identified homeostatic (Hom-MG) and activated (Act-MG) microglial states, concluding that CHA promotes a protective microglial transition. However, we detected significant enrichment of SAT signature genes within the CHA-treated versus control microglia DEGs and in distinct microglial UMAP regions (Supplementary Fig. 14a), suggesting potential confounding by dissociation artifacts.

**Fig. 6:**
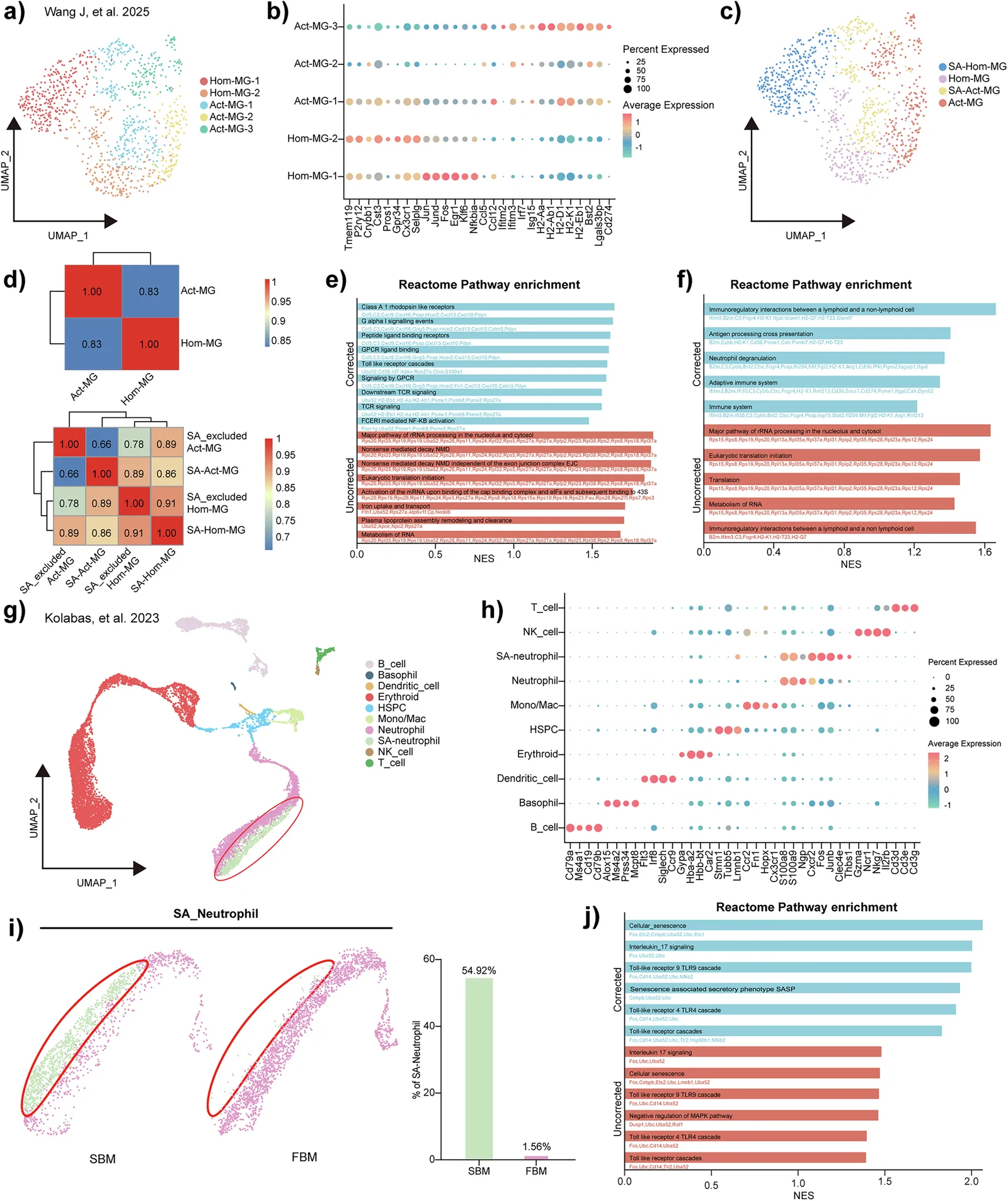
Retrospective correction of published datasets using tissue-specific SAT signatures. **a** Reproduction of microglia clusters from Wang et al. (2025) showing homeostatic (Hom-MG) and activated (Act-MG) subpopulations. **b** Dot plot showing expression of representative marker genes across identified microglial subclusters. Dot size indicates the percentage of cells expressing the gene; color intensity represents average expression. **c** UMAP visualization of SAT signature distribution within microglial populations, color-coded as: homeostatic microglia (Hom-MG), homeostatic microglia expressing SATs (SA-Hom-MG), activated microglia (Act-MG), and activated microglia expressing SATs (SA-Act-MG). **d** Spearman correlation analysis for corrected microglial cells. Top: Correlation analysis of the original total transcription profile, depicting pre-identified active microglia (Act-MG) and homeostatic microglia (Hom-MG). Bottom: Correlation analysis of the total transcription profile after in silico correction of SA cells. This panel presents four groups based on SAT presence: SA cell-excluded homeostatic microglia (SA-excluded-Hom-MG), homeostatic microglia expressing SATs (SA-Hom-MG), SA cell-excluded activated microglia (SA-excluded-Act-MG), and activated microglia expressing SATs (SA-Act-MG). **e** Reactome pathway enrichment analyses were performed on DEGs from Act-MG versus Hom-MG before (annotated in red) and after SAT-based correction (annotated in blue). NES are shown for selected significantly upregulated pathways. **f** Reactome pathway enrichment of microglial DEGs from control versus chlorogenic acid (CHA)-treated groups before (annotated in red) and after SAT-based correction (annotated in blue). NES are shown for selected significantly upregulated pathways. **g** Re-analysis of Kolabas et al. (2023) SBM data showing major immune and hematopoietic cell populations. **h** Dot plot displaying marker gene expression across SBM cell clusters. Dot size indicates the percentage of cells expressing the gene; color intensity represents average expression level. **i** UMAP visualization comparing neutrophil subpopulations in SBM (left) versus femur bone marrow (FBM). SAT-expressing neutrophils (green) and homeostatic neutrophils (pink) are highlighted.** j** Reactome pathway enrichment analysis of DEGs from SBM versus FBM neutrophils before (annotated in red) and after SAT-based correction (annotated in blue). NES are shown for selected significantly upregulated pathways.

To address this, we reproduced the original microglia clustering from this published dataset (Fig. 6a, b). Application of our brain-specific SAT signature revealed distinct subpopulations of stress-altered cells within both the homeostatic (SA-Hom-MG, primarily in Hom-MG-1) and activated (SA-Act-MG, primarily in Act-MG-1) microglial clusters, indicating the presence of stress-induced transcriptional noise (Fig. 6a, c). To quantify this effect, we performed correlation analysis of global transcriptional profiles. The original Hom-MG and Act-MG clusters showed moderate distinction (r = 0.83). Notably, the SA-Act-MG subpopulation exhibited high similarity to Hom-MG (r = 0.89), suggesting that a subset of stress-altered homeostatic microglia had been misclassified as activated. Indeed, computational removal of SA-cells enhanced the transcriptional distinction between the resulting SA-excluded-Act-MG and SA-excluded-Hom-MG (r = 0.78, Fig. 6d). Furthermore, in silico removal of SA-microglia substantially enhanced the biological distinction between Hom-MG and Act-MG populations, with pathway analysis revealing more specific functional profiles for each population (Fig. 6e). Consistent with this refinement, SAT correction also strengthened the enrichment of immune-related pathways in CHA-treated microglia, further strengthening the distinctive pharmacological effects of CHA on microglia (Fig. 6f).

Despite the limited availability of SBM scRNA-seq data, we sought to validate our SAT gene signature using a recently published study^10^. Utilizing raw data and marker genes from this study, we reproduced the clustering of SBM cells (Fig. 6g, h). Application of the SBM-specific SAT signature revealed distinct separation within the neutrophil population, indicating the presence of stress-induced alterations (Fig. 6i, highlighted with red circles), and the SA-neutrophils were enriched with SAT signatures (Supplementary Fig. 14b). To further confirm that these SA-neutrophils were a consequence of enzymatic dissociation, we compared SBM and femur bone marrow (FBM) neutrophils, as the FBM samples were generated using mechanical dissociation. Consistent with our hypothesis, SA-neutrophils were primarily found in SBM (54.92%), with only a marginal presence (1.56%) in FBM (Fig. 6i).

Given the study’s focus on the distinct profile of SBM compared to marrow from other bones^10^, we investigated whether ex vivo artifacts obscured genuine differential gene expression between SBM and FBM neutrophils. Following in silico removal of SA-neutrophils, we re-performed the DEG analysis reported in the original paper and ran GSEA analysis to evaluate the robustness of the original conclusions. Notably, since both FBM and SBM samples in the original study were well processed, the in silico correction served to validate and amplify the initial findings. Key pathway signatures exhibited increased NES magnitudes after artifact removal, thereby strengthening the validity of the reported SBM–FBM differences (Fig. 6j). Furthermore, this refined analysis uncovered a previously masked senescence-associated secretory phenotype (SASP) specifically in SBM neutrophils, suggesting other aspects of their tissue-specific function.

## Discussion

The accurate interpretation of single-cell transcriptomic data is critical for unraveling complex biological processes. However, dissociation-induced stress artifacts lead to widespread transcriptional alterations that severely confound biological interpretation, particularly in sensitive neuroimmune tissues. Here, we address this pervasive challenge with a comprehensive, two-pronged strategy that eliminates these artifacts. By integrating an optimized experimental protocol with a random forest-based computational tool based on tissue-specific SAT signatures, we provide both a preventative and a corrective framework, thereby promoting data fidelity, particularly in neuroimmune profiling.

The transcriptional noise introduced by tissue dissociation has been a known confounder, with immediate early gene (IEG) induction serving as a common indicator across cell types^5,12^. However, our systematic analysis reveals a critical limitation of such universal markers: they are not uniformly present across all cell types and often fail to clearly segregate artifact-prone cells from homeostatic populations. We observed a differential sensitivity to dissociation stress across cell types. Populations such as microglia, monocytes/macrophages, and SMCs exhibited pronounced SAT-driven subclustering, consistent with their known roles as rapid responders to environmental stimuli^32,33^. In contrast, neuronal artifacts reported in region-specific studies^6,13^ were minimal in our whole-brain analysis, likely attributable to our optimized, gentler dissociation protocol. This indicates that the manifestation of SAT is not merely cell-type-specific but is also profoundly influenced by experimental rigor, arising from a complex interplay of stress factors and intrinsic cellular vulnerability. While elevated temperatures common in enzymatic protocols appeared to be a primary driver of the SAT signatures we captured, the complex nature of tissue dissociation suggests a multifactorial origin for these transcriptional alterations. Therefore, we adopted the encompassing term “stress-altered transcription” to reflect this broader stress response, rather than focusing solely on temperature.

While our optimized experimental protocol effectively generates high-fidelity new datasets, a robust computational strategy is indispensable for the vast repository of existing data. Conventional approaches that rely on filtering cells based on universal artifact markers (e.g., IEGs or heat shock proteins)^5,12^ lack the precision to accurately distinguish dissociation artifacts from genuine biology. This is because they fail to account for the cell-type and tissue-specific nature of the dissociation stress response, as evidenced by the inconsistent performance of these universal markers across different lineages.

It should be noted that the use of the OPT condition as “stress-free” baseline in this study has its limitations. The OPT condition contains transcriptional and translational inhibitors that not only suppress the stress-induced artifacts, but may also induce active transcriptional/translational inhibitions, thereby confounding its ability to faithfully reflect the in vivo transcriptional state. To further examine whether the OPT condition loses biological signals due to such inhibition, we integrated Brain_OPT, Dounce_In (with inhibitor, GSE152183), and Dounce_No (without inhibitor) for transcriptional similarity analysis (Supplementary Fig. 15). As shown in the figure, they exhibited a high correlation (0.994), indicating that the inhibitors had minimal impact on cells. Brain_OPT showed similar correlation scores with Dounce_In (0.91) and Dounce_No (0.908), suggesting that although the OPT condition may lose some in vivo signals due to transcriptional/translational inhibition, its overall transcriptional profile remains largely comparable to the Dounce baseline. We acknowledge that the most definitive validation would require direct comparison with in situ hybridization (ISH) or snRNA-seq data in future studies.

Our random forest-based computational tool directly addresses this limitation. It is trained not on a universal gene set, but on tissue-specific SAT signatures that were systematically derived from the most vulnerable cell populations within each tissue. The necessity of this tissue-tailored approach is powerfully demonstrated by our analysis of the SBM. In this critical neuroimmune niche, we defined a unique SAT signature that was distinct from the signature derived from brain tissue. This fundamental divergence underscores that the dissociation stress response is not a generic program but is fundamentally shaped by the unique cellular and molecular microenvironment of each tissue. Our tool enables high diagnostic accuracy with a positive prediction value of 90%, negative prediction value of 98%. It should be noted that high PPV and NPV values have their limitations, as cells in specific pathological states (e.g., exA) are rare within the overall cell population, making it difficult to obtain a more balanced dataset. Considering the prediction value can be affected by class imbalance, we here report the class-balanced metrics, including F1 scores and Matthews correlation coefficients (Supplementary Data 9).

However, our results demonstrate that our computational framework successfully integrates random forest classification and differential expression analysis to define a comprehensive, multi-gene stress-associated transcriptional (SAT) signature for each tissue. We acknowledge that we did not test the SAT across independent tissues beyond brain and SBM that also require enzymatic dissociation (e.g., tumor). Moreover, due to the scarcity of publicly available SBM datasets, only one external validation set^10^ was available. Therefore, we strongly recommend that readers follow the experimental and computational framework established in this study to derive their own tissue-specific SAT signatures, as SAT signatures may exhibit tissue specificity, likely reflecting differences in cellular composition, extracellular matrix, and stress responses across tissues. Consistent with this view, the brain and SBM SAT signature genes show only partial overlap.

It should be noted that our computational framework and the corresponding SAT signatures are not primarily intended to “discover” new cell populations. Instead, they serve as a robust and comprehensive “probe” for systematically identifying and quantifying dissociation-induced transcriptional changes in single-cell data. Their value lies in their integrative and tissue-specific nature, rather than in any claimed improvement in sensitivity over the original exA markers.

Building on this demonstration of tissue specificity, we directly addressed a major analytical challenge: the potential overlap between dissociation-induced SAT and genuine disease-associated stress pathways. Critically, we attempted to distinguish between dissociation-induced pseudo-activation and true disease-driven activation in microglia using the glioma dataset. Our analysis indicates that the pseudo-activated state induced by dissociation stress (SA-Act-MG) is transcriptionally distinct from the true activated state driven by the tumor microenvironment (SA-excluded-Act-MG), with a Spearman correlation of 0.66 between their transcriptomic profiles (Fig. 6d). This suggests that although the two states share certain core stress-response pathways, their overall transcriptional programs are substantially different. Our SAT model was specifically trained to capture subtle patterns distinguishing SA cells from homeostatic states, thereby identifying dissociation-induced pseudo-activation rather than all forms of activation. When applied to published neuroinflammatory datasets, this model can distinguish the“ground truth” of dissociation artifacts from the transcriptional landscape of disease with high confidence. Our re-analysis demonstrates that this approach successfully mitigates confounding pro-inflammatory signals attributable to sample preparation, while preserving and clarifying the disease-relevant biological signatures. This indicates that our method provides a powerful strategy to enhance biological interpretability, even in complex disease contexts where stress pathways are active.

However, we strongly recommend that researchers who wish to apply our SAT model to disease datasets consider two complementary strategies. First, when available, integrate spatial information, cellular distribution patterns, and clinical annotations to inform interpretation. Second, perform differential expression and functional enrichment analyses before and after removal of SAT-positive cells to assess potential alterations in key disease pathways and to evaluate whether filtering is overly aggressive.

Our study establishes a foundational framework and toolkit for addressing dissociation artifacts, transitioning the field from merely acknowledging this problem to actively resolving it. While our tissue-level approach provides a robust and actionable path, it also paves the way for future refinements, such as delineating SAT signatures at the level of cell subtypes and integrating our method with other approaches for comprehensive data purification.

By open-sourcing our SAT toolkit, we aim to catalyze this next stage of development. We envision that it will empower the community to build tailored solutions for diverse tissues and conditions, driving a community-wide refinement of artifact correction standards. In conclusion, our work ensures that single-cell transcriptomics can more faithfully capture the authentic in vivo biology of neuroimmune systems and beyond, thereby empowering more precise and reliable discoveries in health and disease.

## Methods

### Mice

C57BL/6 J mice (stock No. 000664) were purchased from The Jackson Laboratory and housed under specific pathogen-free (SPF) conditions in the Animal Facility Center of Chongqing International Institute for Immunology (CIII) at 23-25 °C with a 12-12 light-dark cycle. Mice were fed ad libitum with standard chow and water. All scRNA-seq studies were performed with 3-month-old male mice. Mice were randomly allocated to control and OPT groups. All experiments were performed in compliance with institutional guidelines and received ethical approval from the Institutional Animal Care and Use Committee (IACUC) of Chongqing International Institute for Immunology. We have complied with all relevant ethical regulations for animal use in this study.

### Preparation of inhibitor cocktail

The inhibitor cocktail, adapted from a previously described protocol^4^, comprised three components: Actinomycin D (Selleck, Cat. #S8964), Triptolide (Selleck, Cat. #S3604), and Anisomycin (Selleck, Cat. #S7409). Stock solutions were prepared as follows: Actinomycin D was reconstituted in DMSO (Invitrogen, Cat. #D12345) to 5 mg/mL; Triptolide was dissolved in DMSO to 10 mM; Anisomycin was reconstituted in DMSO to 10 mg/mL. All stock solutions were aliquoted and stored protected from light. Actinomycin D and Triptolide were stored at −20 °C, and Anisomycin was stored at 4 °C. Stock solutions were used within one week of reconstitution. These inhibitors were added to the perfusion buffer, digestion buffer, and MACS binding buffer in the protocol accordingly.

### Preparation of key buffers for single-cell isolation

Perfusion buffer: Ice-cold PBS (without Ca^2+^, Mg^2+^ or Phenol Red) was supplemented with Actinomycin D (1:1000 dilution from stock) and Triptolide (1:1000 dilution from stock). Inhibitors were added immediately before perfusion; the buffer was stored on ice and protected from light.

Digestion buffer: A papain dissociation system was used according to the manufacturer’s instructions (Worthington, Cat. #LK003150) for brain tissue. For SBM, complete RPMI medium was used, which consisted of RPMI (Gibco, Cat. # 11875093) supplemented with 2% FBS, 1% L-glutamine (Gibco, Cat. #25030081), 1% penicillin–streptomycin (Gibco, Cat. #15140122), 1% sodium pyruvate (Gibco, Cat. #11360070), 1% non-essential amino acids (Gibco, Cat. #11140050) and 1.5% HEPES (Gibco, Cat. #15630080). Immediately before use, 1 mL of complete RPMI medium was supplemented with collagenase VIII (Sigma, Cat. #C2139) and DNase I (Sigma, Cat. #DN25) to final concentrations of 1 mg/mL and 50 U/mL, respectively.

All digestion buffer was supplemented with Actinomycin D (1:1000 dilution from stock), Triptolide (1:1000 dilution from stock), and Anisomycin (1:368.5 dilution from stock) immediately before incubation at 37 °C for enzymatic digestion.

MACS binding buffer: The MACS binding buffer for dead cell removal was prepared per the manufacturer’s instructions (Miltenyi, Cat. #130-090-101) and supplemented with Actinomycin D (1:1000 dilution from stock), Triptolide (1:1000 dilution from stock), and Anisomycin (1:368.5 dilution from stock) immediately before adding digested cells.

### Single-cell isolation for brains

The single-cell isolation method was adapted from a previously described protocol^4^, with optimization for enhanced efficiency and cost-effectiveness. Mice were anesthetized and intracardially perfused with the perfusion buffer, with or without the inhibitors. Brains were rapidly dissected and immersed in 10 mL ice-cold PBS, protected from light, containing either no inhibitors (CTL group) or the inhibitor cocktails (Actinomycin D, 1:1000; Triptolide, 1:1000) (OPT group). Brains were then transferred to a Petri dish containing papain digestion buffer (Worthington, Cat. # LK003150) with or without the inhibitors (Actinomycin D, 1:1000; Triptolide, 1:1000; Anisomycin, 1:368.5) on ice. The brain tissue was minced into small pieces (<10 mm³), transferred to a 50 mL Falcon tube, and incubated at 37 °C with gentle agitation for 20 minutes. The digested brain mixture was triturated to further dissociate cells, followed by centrifugation at 4 °C and 500 *g* for 5 minutes. The cell pellet was resuspended in EBSS (supplemented with recombinant albumin-ovomucoid and DNase) with or without inhibitors and centrifuged at 4 °C, 500 *g* for 5 minutes. Cells were then resuspended in EBSS, filtered through a 70 µm cell strainer, and centrifuged at 4 °C, 500 *g* for 5 minutes. The resulting cell pellet was resuspended with pre-cooled 30% Percoll solution (9 mL Percoll + 1 mL 10X PBS + 23 mL 1X PBS) and centrifuged at 4 °C, 700 *g* for 20 minutes (without brakes). After carefully removing the myelin-containing supernatant, cells were resuspended in PBS, and dead cells were removed using a Dead Cell Removal kit (Miltenyi, Cat. #130-090-101) according to the manufacturer’s instructions. MS columns in the magnetic separator were used with or without inhibitors, depending on the experimental group. The resulting live cells were then centrifuged and resuspended in PBS with 0.2% BSA for subsequent single-cell library construction.

### Single-cell isolation for SBM

Mice were anesthetized and intracardially perfused with perfusion buffer, with or without inhibitors. Calvaria were rapidly dissected, and the meninges and other adherent tissues were meticulously removed. The calvaria were then transferred to ice-cold digestion buffer (no inhibitors were added at this step due to the low temperature), and finely minced using pre-cooled scissors into pieces <2 mm × 2 mm. Once minced, an inhibitor cocktail was added, and the samples were incubated at 37 °C for 20 minutes to facilitate digestion. Digestion was quenched by adding an equal volume of RPMI (supplemented with 10% FBS), and the samples were centrifuged at 500 *g* for 5 minutes at 4 °C. Red blood cells were lysed by incubating the cell pellet with ACK lysis buffer (Gibco, Cat. #A1049201) on ice for 3 minutes. The resulting cell suspension was filtered through a 40 µm cell strainer and centrifuged at 300 g for 10 minutes at 4 °C. Cells were then resuspended in MACS binding buffer, with or without inhibitors, and dead cell removal microbeads were added according to the manufacturer’s instructions (Miltenyi, Cat. #130-090-101). Labeled cells were separated using a MS column, and viable cells were collected, centrifuged, and resuspended in PBS (with 0.2% BSA) for library generation.

### BV2 microglia cell culture and preparation

BV2 microglial cells (Serial: GNM45, National Collection of Authenticated Cell Cultures) were maintained in DMEM supplemented with 10% fetal bovine serum and 1% penicillin-streptomycin at 37 °C with 5% CO₂. Before the heat-shock experiment, cells were seeded in 6-well plates until confluency. Six hours prior to treatment, the medium was replaced to synchronize cellular states.

### BV2 microglia cell heat-shock experiment

BV2 cells were divided into three experimental groups. RT (room temperature) group: Cells were transferred to the biosafety cabinet and returned to the 37 °C incubator for 2 hours; HS (heat-shock) group: Cells were transferred to a 42 °C incubator for 2 hours; HSIN (heat-shock with inhibitors) group: Cells were supplemented with an inhibitor cocktail containing Actinomycin D (1:1000), Triptolide (1:1000), and Anisomycin (1:368.5) prior to 2-hour incubation at 42 °C. Following treatment, cells were washed twice with PBS and either collected for RT-qPCR analysis (performed using SYBR ® Green Real-time PCR Master Mix, with the corresponding primers that listed in Supplementary Data 10) or snap-frozen in TRIzol (1 mL) in liquid nitrogen and sent to Novogene for bulk RNA-seq.

### RNA extraction and library construction from BV2 cells

Total RNA was extracted using a standard TRIzol-based protocol. Briefly, cell lysates were mixed with chloroform (200 μL), vigorously shaken for 15 seconds, and incubated at room temperature for 2–3 minutes. Phase separation was achieved by centrifugation at 12,000 × *g* for 10–15 minutes at 4 °C. The upper aqueous phase containing RNA was carefully transferred to a new tube, and RNA was precipitated by adding an equal volume of isopropanol, followed by incubation at room temperature for 10 minutes and centrifugation at 12,000 × *g* for 10 minutes at 4 °C. The RNA pellet was washed with 1 mL of 75% ethanol (DEPC-treated), centrifuged at 12,000 × *g* for 3 minutes at 4 °C, and air-dried for 2–3 minutes. Purified RNA was dissolved in RNase-free water, assessed for quality, and stored at −80 °C.

For transcriptome sequencing, total RNA samples passing quality control were subjected to mRNA enrichment using poly-T magnetic beads. The enriched mRNA was fragmented, and double-stranded cDNA was synthesized using random hexamer primers. Sequencing libraries were constructed through end repair, A-tailing, adapter ligation, size selection, and PCR amplification. Library quality was assessed by measuring concentration (Qubit/RT-PCR) and size distribution (Bioanalyzer). Qualified libraries were pooled based on effective concentration and sequenced on an Illumina platform using Sequencing-by-Synthesis chemistry. Raw sequencing reads were processed with fastp to remove adapter sequences, reads containing >10% unknown bases, and low-quality reads (>50% bases with Q ≤ 5). High-quality clean reads were obtained for downstream analysis, with standard quality metrics including Q20, Q30, and GC content calculated for quality assessment.

### Single-cell RNA sequencing (scRNA-seq)

Library preparation and sequencing were performed by Novogene Co., Ltd. using the 10X Genomics Chromium Platform. Briefly, single-cell suspensions were loaded onto a 10X Chromium Controller (10X Genomics) at a concentration of 1000 cells/uL, targeting a capture of 10,000 cells per well. Gel Beads-in-Emulsion (GEMs) droplets were generated, and scRNA-seq libraries were constructed using Chromium Next GEM Chip K Single Cell Kit (10X Genomics) and Chromium Next GEM Single Cell 5’ V2 GEM, Library & Gel Bead Kit (10X Genomics). Each GEM underwent a reverse transcription reaction to generate single-cell cDNA, when each encapsulated cell was labeled with a unique 12-nt cell barcode, and each transcript was labeled with a unique molecular identifier (UMI). Amplified cDNA was used for library preparation, consisting of fragmentation, end repair, A-tailing, adaptor ligation, and sample index PCR according to the manufacturer’s instructions. Final libraries were sequenced on a NovaSeq X Plus Sequencing System (Illumina).

### scRNA-seq data analysis

#### scRNA-seq analysis software and packages

All scRNA-seq data analysis was conducted using R (v4.2.0) (https://www.r-project.org) and Python (v3.10.13). The primary analysis utilized the Seurat package (v4.2.0). Doublets were detected and removed using DoubletFinder (v2.0.3). Data visualization was performed with scCustomize (v3.0.0). The VennDiagram (v1.7.3), ggplot2 (v3.4.0), and ComplexHeatmap (v2.14.0) packages were employed to generate Venn diagrams, volcano plots, and heatmaps, respectively. Gene set enrichment analysis (GSEA) was performed using the fgsea package (v1.28.0), and results were visualized with ggpubr (v0.5.0). In this study, batch effects were corrected using Harmony (v0.1.1).

#### Count matrix generation and quality control

The scRNA-seq data were demultiplexed, aligned, and processed into count matrices using the Cell Ranger software (v7.0.0) with default parameters. The processing pipeline included alignment to the mm10 reference genome (provided by 10X Genomics) and incorporation of intronic regions (see 10X Genomics documentation for details https://github.com/10XGenomics/cellranger). Raw gene expression matrices were generated by directly importing Cell Ranger outputs into Seurat (v4.2.0).

For brain samples, 19,034 cells passed quality control (QC) thresholds based on the following criteria: exclusion of genes detected in fewer than three cells; a minimum threshold of 500 unique genes per cell; exclusion of cells with UMI counts exceeding 12,000; and removal of cells with mitochondrial read proportions >10%. Doublets were identified using DoubletFinder (v2.0.3), and 462 doublets were subsequently removed.

For SBM samples, 21,975 cells passed QC thresholds: genes detected in fewer than three cells were excluded; cells were retained if expressing 200–6000 unique genes; cells with UMI counts exceeding 20,000 or mitochondrial read proportions >10% were excluded. DoubletFinder (v2.0.3) identified 1505 doublets, which were then manually removed.

#### Data processing and annotation

Brain samples: following quality control, raw count data were processed using the Seurat package for dimensionality reduction, clustering, and annotation. First, the SCTransform function was applied to normalize and scale the data while regressing out UMI counts per cell and mitochondrial gene percentages. Principal component analysis (PCA) was performed for dimensionality reduction, with the optimal number of principal components (PCs) selected based on the inflection point (“elbow”) of the ElbowPlot. A shared nearest neighbor (SNN) graph was constructed using the top 30 PCs, and clusters were identified via the Louvain clustering algorithm with a resolution parameter empirically optimized for the dataset.

Cell type annotation was performed by manually identifying differentially expressed marker genes across clusters. DEGs were detected using the FindAllMarkers function (Wilcoxon rank-sum test, adjusted *p*-value < 0.05 with Bonferroni correction, fold-change >1.5). Annotation was further guided by canonical markers from published single-cell datasets^4,34–36^. Subclustering was performed for microglia, mono/mac and SMCs. Subset data were reprocessed using SCTransform, scaled, and subjected to PCA. The top 20 PCs were used for Louvain clustering and UMAP visualization.

The definition and annotation of exAM/exA cells in the brain were adopted from Marsh et al.^4^. For exAM, unsupervised subclustering of microglia was performed to identify the distinct clusters, then the representative exAM gene signature was used as the marker for annotation of exAM. For exA cells, subclustering was performed for each cell type, and the exAM gene signature was used as the marker for the annotation of exA cells.

SBM samples: A similar analytical pipeline was applied to SBM samples, with modifications. Data normalization was performed using the NormalizeData function, followed by identification of the top 2000 variable features. Data were scaled, and UMI counts per cell and cell cycle effects were regressed out. Neutrophils, mono/mac, and HSPCs were isolated for downstream analysis. Subset analyses followed the same workflow as described for brain samples.

#### Differential expression analysis

Differential expression analysis was performed to identify potential signature genes of SA-clusters in brain and SBM samples. Previously defined exA genes^4^ were utilized for clustering exA-clusters and homeo-clusters within brain sub-clusters (microglia, monocytes/macrophages, SMCs) and SBM sub-clusters (neutrophils, monocytes/macrophages, HSPCs). The FindMarkers function was employed to identify DEGs between exA-cluster and homeo-cluster across sub-clusters, using thresholds of Benjamini-Hochberg corrected *p*-value < 0.05 and log2FC > 0.5. Genes exhibiting an average log2-fold change > 1 across comparisons were designated as signature genes. Volcano plots visualized differentially expressed genes within each sub-cluster, with exA genes highlighted in black and signature genes in red. Signature genes were integrated with variable features to guide re-clustering of brain and SBM sub-clusters, followed by manual annotation of HA-clusters and homeo-clusters. Three comparative DEG analyses were conducted across sub-clusters: (i) CTL_1 vs OPT_1 (full dataset), (ii) CTL_2 vs OPT_2 (exA-cell excluded), and (iii) CTL_3 vs OPT_3 (HA-cell excluded). In SBM sub-clusters, two comparisons were executed: (i) CTL_1 vs OPT_1 (full dataset) and (ii) CTL_3 vs OPT_3 (HA-cell excluded). Hierarchically clustered heatmaps visualized distinct expression patterns among exA genes, SA signature genes, and the top 50 DEGs across experimental conditions.

#### Pathway enrichment analysis

Gene Set Enrichment Analysis (GSEA) was implemented using the fgsea package (v1.28.0) to evaluate canonical pathways from Reactome, Kyoto Encyclopedia of Genes and Genomes (KEGG), and Gene Ontology (GO) databases, with gene sets containing 10-500 genes to mitigate over-representation bias from extremely small or large gene sets while maintaining biological interpretability. Pathway annotations were obtained from the Molecular Signatures Database (MSigDB) through its mouse collections portal. Significantly enriched pathways (*p*-value < 0.05 and absolute value of NES > 1) were visualized using the ggdotchart function, enabling comparative analysis of enrichment patterns across brain and SBM sub-clusters. For validation with public datasets, significantly enriched pathways (*p*-value < 0.05 and |NES | > 1) were visualized using the ggplot function.

### Quantitative assessment of cluster stability

To evaluate clustering stability, the Adjusted Rand Index (ARI) was computed using the adjusted_rand_score() function from the Scikit-learn package (v1.6.1). Two sets of ARI values—one with SA included and another with SA excluded—were calculated across three clustering algorithms (Louvain, Leiden, and cNMF) for both brain and SBM samples.

### Machine learning model construction and validation

#### Feature selection and model training

For the brain and SBM random-forest (RF) models, the Python package Scikit-learn (v1.6.1) and the RandomForestClassifier() function were employed to train a machine learning model for discriminating between homeostatic cell clusters and SA cell clusters. Development of a computational pipeline involves the following five steps: (i) dataset preprocessing, (ii) feature selection, (iii) model training, (iv) identification of the most important features, and (v) model evaluation.

All cohort data used for training and validation were first processed using LogNormalize from the Seurat package. Subsequently, the data were transformed into a feature matrix X and a label vector Y. The feature matrix X represents gene expression data, while the label vector Y contains the cell type labels, which were encoded using LabelEncoder.

To eliminate redundant features and noise and ensure the classifier only distinguished SA versus homeostatic states rather than cell types. We first identified the common differentially expressed genes (DEGs) between SA and homeostatic cells across different cell types in each tissue sample. Only these common DEGs were used as input features for model training, using normalized expression values. Then, we input the expression matrices of several cell types (including Brain: microglia, Mono/Mac, SMC; SBM: neutrophil, Mono/Mac, HSPC) into the RF model for pre-training, with 80% of the samples assigned to the training set and 20% to the testing set. The RF model was created using the RandomForestClassifier class, with the parameters tuned using a grid search algorithm with five-fold cross-validation, and a random seed set. For the brain model, search space was defined as ‘n_estimators’ [50, 100, 150, 200],‘max_depth’ [None, 10, 20, 30, 40],‘min_samples_split’[2,5,10,20]:,‘min_samples_leaf’[1,2,4,6]:,‘bootstrap’ [True]. The optimal hyperparameters were determined to be:‘bootstrap’ True,‘max_depth’ 20, ‘min_samples_leaf’ 2,‘min_samples_split’ 2,‘n_estimators’ 150. For SBM model, the hyperparameters search space was defined as ‘n_estimators’ [50, 100, 150, 200],‘max_depth’:[None, 10, 20, 30, 40],‘min_samples_split’[2,5,10,20]:,‘min_samples_leaf’[1,2,4,6],‘bootstrap’ [True]. The optimal hyperparameters were determined to be: ‘bootstrap’ True, ‘max_depth’: None, ‘min_samples_leaf’ 1,‘min_samples_split’ 5, ‘n_estimators’ 200.

After model fitting, features were ranked by importance, and the top 50 most significant features were retained. We then identified candidate signature genes by filtering the differentially expressed genes (DEGs) obtained from the FindMarkers() function. DEGs meeting the following criteria were selected: an average log2 fold-change (avg_log2FC) > 0.5 and an adjusted *p*-value < 0.05 using the Bonferroni method. This filtering process yielded 11 candidate signatures across the aforementioned cell types. Additionally, 8 reference exA genes reported by Beth Stevens et al. 4 were included, resulting in a total of 53 unique gene features for RF1 (used to identify SA cells in the brain) and 55 unique gene features for RF2 (used to identify SA cells in SBM). The RF models were then retrained using the same strategy.

#### Model validation

The GSE123025, GSE121654, and GSE152182 cohorts were used as the external independent test sets for RF1, among which the GSE121654 cohort was a cold Dounce dataset composed of homeostatic cells as the negative control. RF2 used GSM5753481 and GSM5753483 as external independent test sets to further evaluate the performance of the RF model. First, the random forest model was trained using a set of SAT signature genes (53 genes for brain, 55 genes for SBM). For each validation dataset, we extracted the normalized expression values of these genes from all cells. These expression values were then used as input to the trained model, which generated a prediction for each cell (SA vs. homeostatic). In each validation dataset, cells were independently classified as SA or homeostatic depending on their original annotation, and we compared the model predictions against these independent annotations and calculated the area under the curve (AUC) to quantify classification performance. To estimate uncertainty, 95% confidence intervals (CIs) for each AUC were computed using 1000 bootstrap resamples. For external validation of the SBM model, a randomly selected subset (50%) of cells from GSM5753481 and GSM5753483 was held out and never used in any downstream application analysis. The remaining 50% of cells were reserved for the retrospective correction demonstration, ensuring complete independence between model validation and application. Corresponding confusion matrices and ROC plots were generated for assessing the performance of the assemblers using the matplotlib (V.3.7.0) and seaborn (V.0.13.2) packages.

### Processing and analysis of public scRNA-seq datasets

#### Microglia comparative analysis

Comparative analysis was performed between microglia from previously published scRNA-seq datasets (Gene Expression Omnibus (GEO) accessions: GSM4605687, GSM4605691, GSM4605695) generated using cold mechanical Dounce homogenization^4^ and ours. Following the quality control criteria, microglia subsets were isolated from our dataset and integrated with external datasets. Batch effect correction was implemented using the Harmony (v0.1.1) package prior to downstream comparative analysis, enabling direct evaluation of protocol-dependent transcriptional responses.

### Retrospective correction of public scRNA-seq datasets

To validate the generalizability and utility of our tissue-specific SA signatures, we applied our computational pipeline to two independent, publicly available scRNA-seq datasets.

#### Analysis of brain dataset

A previously published single-cell dataset of the glioma immune microenvironment was re-analyzed. Data were obtained from the OMIX repository (accession no. OMIX008544) hosted by the China National Center for Bioinformation/Beijing Institute of Genomics, Chinese Academy of Sciences^31^. Raw gene expression matrices were processed using Seurat. Cells were filtered based on the following quality control criteria: cells were retained if they expressed between 500 and 3000 unique genes and had total UMI counts greater than 500; cells with mitochondrial gene content exceeding 20% were excluded. Data normalization, scaling, and integration were performed using the Harmony algorithm. In contrast to our own data, the public datasets have significant batch effects; therefore, we have applied Harmony (v0.1.1) for batch effect correction before analysis. Regression of confounding factors (mitochondrial gene expression, ribosomal gene expression, and cell cycle score) was conducted. Microglia clusters were identified based on canonical marker genes and subsetted for all subsequent analyses.

#### Analysis of SBM dataset

Publicly available single-cell data from naïve calvaria and femur marrow (GEO accessions GSM5753481, GSM5753483)^10^ were downloaded and processed. To ensure independence between model validation and downstream application, the dataset was randomly split into two subsets: 50% of cells were used exclusively for external validation of the SBM random forest model, and the remaining 50% were used for the retrospective correction demonstration described below. Cells were filtered if total UMI counts were below 1000 or above 50,000, or if mitochondrial read percentage was greater than 20%. Neutrophil clusters were identified based on established markers and isolated for further investigation. Differential expression analysis between homeostatic neutrophils and SA-neutrophils was performed, and the top 50 differentially expressed genes (*p*-value < 0.05, log2FC > 0.5) alongside SAT-associated genes were visualized via heatmap.

#### In silico SAT-correction pipeline

For both datasets, the subsetted cell populations (microglia or neutrophils) were scored using their corresponding pre-trained, tissue-specific machine learning model. Cells classified as SAT-positive (SA-cells) were flagged. To generate a corrected dataset, these SA-cells were computationally removed. All downstream comparative analyses, including differential expression and gene set enrichment analysis, were performed between the original (uncorrected) and the SAT-corrected datasets.

### Bulk RNA-seq analysis

Bulk RNA-seq data were generated from three experimental conditions: room temperature (RT), heat shock (HS), and heat shock with inhibitor (HSIN). Raw sequencing reads in FASTQ format were processed using the OmicVerse Python package (v1.6.10). Sequence quality was assessed with FastQC (v0.12.1), and adapter sequences along with low-quality bases were trimmed using TrimGalore (v0.6.10) with standard parameters. High-quality reads were aligned to the mouse GRCm39 reference genome (GENCODE annotation vM10) using HISAT2 (v2.2.1). Gene-level read counts were quantified from the resulting BAM files using FeatureCounts (v2.1.1) for downstream analysis.

Differential expression analysis between experimental conditions was performed using Omicverse and the pyDEG() function (v1.38.3) in Python. The DESeq2 model incorporated the experimental condition as the primary factor. Genes with an adjusted *p*-value (FDR) < 0.05 and an absolute log₂ fold-change > 0.5 were considered differentially expressed. Functional enrichment analysis for Gene Ontology (GO) biological processes and KEGG pathways was conducted on differentially expressed gene sets using the ClusterProfiler package (v4.6.2), with significantly enriched terms defined by an FDR cutoff of 0.05.

### Statistics and Reproducibility

For the scRNA-seq experiment, one technical replicate comprising cells pooled from 3 mice was used. Statistical analyses were performed using R (v4.2.0). Comparisons between two groups were performed using the unpaired two-tailed Student’s *t*-test or Mann-Whitney U test, as appropriate based on data normality. A *p*-value ≤ 0.05 was considered statistically significant for all comparisons. Differential gene expression analyses were conducted using Seurat’s FindMarkers function. Genes with a Benjamini-Hochberg corrected *p*-value < 0.05 and an absolute log₂-fold change > 0.5 were considered significant. Signature genes were further designated as those with an average log₂-fold change > 1 across comparisons. GSEA was performed using the fgsea package (v1.28.0) against Reactome and KEGG gene sets. Significantly enriched pathways were defined by a *p*-value < 0.05 and an absolute NES > 1. Steiger’ Z test was used to evaluate the significance of the Spearman correlation improvements. For the RT-qPCR experiment, 3 biologically independent samples were used. Differences among groups were analyzed by one-way analysis of variance (ANOVA) followed by Tukey’s multiple comparisons test using Graphpad Prism (10.2.3). No statistical predetermination of sample size was done. For in vitro studies (qPCR and bulk RNA-seq), sample size=3 was selected based on prior studies demonstrating it was sufficient for replication without large variation. For scRNA-seq, biological triplicates were used for each sample.

### Reporting summary

Further information on research design is available in the Nature Portfolio Reporting Summary linked to this article.

## Supplementary information

Supplementary information

Description of Additional Supplementary Files

Supplementary Data 1

Supplementary Data 2

Supplementary Data 3

Supplementary Data 4

Supplementary Data 5

Supplementary Data 6

Supplementary Data 7

Supplementary Data 8

Supplementary Data 9

Supplementary Data 10

Supplementary Data 11

Reporting summary

## Data Availability

Raw scRNA-seq FASTQ files, the processed count matrix, and bulk RNA-seq data have been deposited in the Gene Expression Omnibus (GEO) database under accession code (GSE294852). The numerical source data for graphs in this study have been uploaded as Supplementary Data 11. Public single-cell datasets for external independent tests can be accessed by GSE123025 and GSE121654. Public single-cell datasets for microglia with different dissociation methods can be accessed by GEO accessions: GSM4605687, GSM4605691, GSM4605695, GSM152182, GSM152183. Public single-cell datasets for naïve calvaria and femur marrow can be accessed by GEO accessions: GSM5753481, GSM5753483. Public single-cell dataset for glioma immune microenvironment can be accessed by the OMIX repository (accession no. OMIX008544).

## Code availability

Source code and analysis scripts were deposited in a public Zenodo repository https://doi.org/10.5281/zenodo.19393742^37^.
