# Supplementary material for: A two-pronged strategy eliminates dissociation artifacts for high-fidelity neuroimmune single-cell transcriptomics
Source: Commun Biol. 2026 Jul 6;9:1294. doi: 10.1038/s42003-026-10609-x (PMC13642925; doi:10.1038/s42003-026-10609-x)
Supplement: Supplementary file 1 [file 42003_2026_10609_MOESM14_ESM.pdf]

nature portfolio

Corresponding author(s): Yiguo Qiu
Last updated by author(s): Jun 12, 2026

Reporting Summary

Nature Portfolio wishes to improve the reproducibility of the work that we publish. This form provides structure for consistency and transparency in reporting. For further information on Nature Portfolio policies, see our Editorial Policies and the Editorial Policy Checklist.

Statistics

For all statistical analyses, confirm that the following items are present in the figure legend, table legend, main text, or Methods section.

n/a	Confirmed	
☐	☒	The exact sample size (*n*) for each experimental group/condition, given as a discrete number and unit of measurement
☐	☒	A statement on whether measurements were taken from distinct samples or whether the same sample was measured repeatedly
☐	☒	The statistical test(s) used AND whether they are one- or two-sided<br>*Only common tests should be described solely by name; describe more complex techniques in the Methods section.*
☐	☒	A description of all covariates tested
☐	☒	A description of any assumptions or corrections, such as tests of normality and adjustment for multiple comparisons
☐	☒	A full description of the statistical parameters including central tendency (e.g. means) or other basic estimates (e.g. regression coefficient) AND variation (e.g. standard deviation) or associated estimates of uncertainty (e.g. confidence intervals)
☐	☒	For null hypothesis testing, the test statistic (e.g. *F*, *t*, *r*) with confidence intervals, effect sizes, degrees of freedom and *P* value noted<br>*Give P values as exact values whenever suitable.*
☒	☐	For Bayesian analysis, information on the choice of priors and Markov chain Monte Carlo settings
☒	☐	For hierarchical and complex designs, identification of the appropriate level for tests and full reporting of outcomes
☒	☐	Estimates of effect sizes (e.g. Cohen's *d*, Pearson's *r*), indicating how they were calculated

Our web collection on statistics for biologists contains articles on many of the points above.

Software and code

Policy information about availability of computer code

Data collection	Cell Ranger software (v7.0.0) were used for raw scRNA-seq data processing. DoubletFinder (v2.0.3) were used to identify doublets in snRNA-seq. scCustomize (v3.0.0), VennDiagram (v1.7.3), ggplot2 (v3.4.0), and ComplexHeatmap (v2.14.0), ggpubr (v0.5.0) and GraphPad Prism (10.2.3) were used for data visualization. Scikit-learn package (v1.6.1) were used for Adjusted Rand Index computation.<br>Bulk RNA-seq data were processed with OmicVerse Python package (v1.6.10), FastQC (v0.12.1),TrimGalore (v0.6.10), HISAT2 (v2.2.1) and FeatureCounts (v2.1.1).
Data analysis	Seurat package (v4.2.0) were primarily used for analysis. fgsea package (v1.28.0) were used for Gene set enrichment analysis. Harmony (v0.1.1) were used for batch effects correction. Python package Scikit-learn (v1.6.1), RandomForestClassifier() function, Seurat package (v4.2.0) LogNormalize and LabelEncoder were used for machine learning analysis. matplotlib (V.3.7.0) and seaborn(V.0.13.2) packages were used for ROC plot generation and performance assessment.<br>Omicverse and pyDEG() function (v1.38.3) were used for Differential expression analysis in Bulk RNA-seq and ClusterProfiler package (v4.6.2) were used for its pathway enrichment.

For manuscripts utilizing custom algorithms or software that are central to the research but not yet described in published literature, software must be made available to editors and reviewers. We strongly encourage code deposition in a community repository (e.g. GitHub). See the Nature Portfolio guidelines for submitting code & software for further information.

Data

Policy information about availability of data

All manuscripts must include a data availability statement. This statement should provide the following information, where applicable:

- Accession codes, unique identifiers, or web links for publicly available datasets
- A description of any restrictions on data availability
- For clinical datasets or third party data, please ensure that the statement adheres to our policy

Raw scRNA-seq FASTQ files, the processed count matrix and Bulk RNA-seq data have been deposited in the Gene Expression Omnibus (GEO) database under accession code (GSE294852). The numerical source data for graphs in this study has been uploaded as Supplementary Data 11.
Public Single cells datasets for external independent test can be accessed by GSE123025 and GSE121654.
Public Single cell datasets for microglia with different dissociation methods can be accessed by GEOaccessions: GSM4605687, GSM4605691, GSM4605695 GSM152182,GSM152183
Public Single cell datasets for naïve calvaria and femur marrow can be accessed by GEO accessions: GSM5753481, GSM5753483.
Public single cell dataset for glioma immune microenvironment can be accessed by OMIX repository (accession no. OMIX008544).
Source code and analysis scripts can be accessed by Zenodo repositoty (doi.org/10.5281/zenodo.19393742)

Research involving human participants, their data, or biological material

Policy information about studies with human participants or human data. See also policy information about sex, gender (identity/presentation), and sexual orientation and race, ethnicity and racism.

Reporting on sex and gender	No human participants, their data, or biological material are involved in this study
Reporting on race, ethnicity, or other socially relevant groupings	No human participants, their data, or biological material are involved in this study
Population characteristics	No human participants, their data, or biological material are involved in this study
Recruitment	No human participants, their data, or biological material are involved in this study
Ethics oversight	No human participants, their data, or biological material are involved in this study

Note that full information on the approval of the study protocol must also be provided in the manuscript.

Field-specific reporting

Please select the one below that is the best fit for your research. If you are not sure, read the appropriate sections before making your selection.

☒ Life sciences ☐ Behavioural & social sciences ☐ Ecological, evolutionary & environmental sciences

For a reference copy of the document with all sections, see nature.com/documents/nr-reporting-summary-flat.pdf

Life sciences study design

All studies must disclose on these points even when the disclosure is negative.

Sample size	No statistical predetermination of sample size was done. For in vitro studies(qPCR and bulk RNA-seq), sample size=3 was selected basing on prior studies demonstrated it was sufficed for sufficient replication without large variation. For scRNA-seq, biological triplicates were used for each sample.
Data exclusions	No data was excluded in this study.
Replication	Experiments were done in triplicate the least unless specified otherwise. The costly scRNA-seq experiments were done only once, but each experiment consists of multiple biological replicates (n=3)
Randomization	Mice were randomly allocated to control and OPT groups.
Blinding	Blinding was not relevant to these studies.

Behavioural & social sciences study design

All studies must disclose on these points even when the disclosure is negative.

Study description

Research sample

Sampling strategy

Data collection

Timing

Data exclusions

Non-participation

Randomization

Ecological, evolutionary & environmental sciences study design

All studies must disclose on these points even when the disclosure is negative.

Study description

Research sample

Sampling strategy

Data collection

Timing and spatial scale

Data exclusions

Reproducibility

Randomization

Blinding

Did the study involve field work? ☐ Yes ☐ No

Field work, collection and transport

Field conditions

Location

Access & import/export

Disturbance

Reporting for specific materials, systems and methods

We require information from authors about some types of materials, experimental systems and methods used in many studies. Here, indicate whether each material, system or method listed is relevant to your study. If you are not sure if a list item applies to your research, read the appropriate section before selecting a response.

Materials & experimental systems

n/a	Involved in the study
☒	☐ Antibodies
☐	☒ Eukaryotic cell lines
☒	☐ Palaeontology and archaeology
☐	☒ Animals and other organisms
☒	☐ Clinical data
☒	☐ Dual use research of concern
☒	☐ Plants

Methods

n/a	Involved in the study
☒	☐ ChIP-seq
☒	☐ Flow cytometry
☒	☐ MRI-based neuroimaging

Antibodies

Antibodies used	
Validation	

Eukaryotic cell lines

Policy information about cell lines and Sex and Gender in Research

Cell line source(s)	BV2 microglial cells- Serial: GNM45, from National Collection of Authenticated Cell Cultures-female
Authentication	Not authenticated
Mycoplasma contamination	Not tested
Commonly misidentified lines (See ICLAC register)	No commonly misidentified lines

Palaeontology and Archaeology

Specimen provenance	
Specimen deposition	
Dating methods	

☐ Tick this box to confirm that the raw and calibrated dates are available in the paper or in Supplementary Information.

Ethics oversight	

Note that full information on the approval of the study protocol must also be provided in the manuscript.

Animals and other research organisms

Policy information about studies involving animals; ARRIVE guidelines recommended for reporting animal research, and Sex and Gender in Research

Laboratory animals	C57BL/6J mice (stock No. 000664) were purchased from The Jackson Laboratory
Wild animals	The study did not involve wild animals
Reporting on sex	Sex-based analyses were not performed. We utilized only males to avoid confounding effects of the estrus cycle.
Field-collected samples	The study did not involve samples collected from the field.
Ethics oversight	Institutional Animal Care and Use Committee (IACUC) of Chongqing International Institute for Immunology

Note that full information on the approval of the study protocol must also be provided in the manuscript.

Clinical data

Policy information about clinical studies

All manuscripts should comply with the ICMJE guidelines for publication of clinical research and a completed CONSORT checklist must be included with all submissions.

Clinical trial registration	
Study protocol	
Data collection	
Outcomes	

Dual use research of concern

Policy information about dual use research of concern

Hazards

Could the accidental, deliberate or reckless misuse of agents or technologies generated in the work, or the application of information presented in the manuscript, pose a threat to:

No	Yes	
☐	☐	Public health
☐	☐	National security
☐	☐	Crops and/or livestock
☐	☐	Ecosystems
☐	☐	Any other significant area

Experiments of concern

Does the work involve any of these experiments of concern:

No	Yes	
☐	☐	Demonstrate how to render a vaccine ineffective
☐	☐	Confer resistance to therapeutically useful antibiotics or antiviral agents
☐	☐	Enhance the virulence of a pathogen or render a nonpathogen virulent
☐	☐	Increase transmissibility of a pathogen
☐	☐	Alter the host range of a pathogen
☐	☐	Enable evasion of diagnostic/detection modalities
☐	☐	Enable the weaponization of a biological agent or toxin
☐	☐	Any other potentially harmful combination of experiments and agents

Plants

Seed stocks	No plant material was used in this study
Novel plant genotypes	No plant material was used in this study
Authentication	No plant material was used in this study

ChIP-seq

Data deposition

- [ ] Confirm that both raw and final processed data have been deposited in a public database such as GEO.
- [ ] Confirm that you have deposited or provided access to graph files (e.g. BED files) for the called peaks.

Data access links<br>*May remain private before publication.*	
Files in database submission	
Genome browser session<br>(e.g. UCSC)	

Methodology

Replicates	
Sequencing depth	
Antibodies	
Peak calling parameters	
Data quality	
Software	

Flow Cytometry

Plots

Confirm that:

- [ ] The axis labels state the marker and fluorochrome used (e.g. CD4-FITC).
- [ ] The axis scales are clearly visible. Include numbers along axes only for bottom left plot of group (a 'group' is an analysis of identical markers).
- [ ] All plots are contour plots with outliers or pseudocolor plots.
- [ ] A numerical value for number of cells or percentage (with statistics) is provided.

Methodology

Sample preparation	
Instrument	
Software	
Cell population abundance	
Gating strategy	

- [ ] Tick this box to confirm that a figure exemplifying the gating strategy is provided in the Supplementary Information.

Magnetic resonance imaging

Experimental design

Design type	
Design specifications	
Behavioral performance measures	

Acquisition

Imaging type(s)

Field strength

Sequence & imaging parameters

Area of acquisition

Diffusion MRI ☐ Used ☐ Not used

Preprocessing

Preprocessing software

Normalization

Normalization template

Noise and artifact removal

Volume censoring

Statistical modeling & inference

Model type and settings

Effect(s) tested

Specify type of analysis: ☐ Whole brain ☐ ROI-based ☐ Both

Statistic type for inference

(See Eklund et al. 2016)

Correction

Models & analysis

n/a	Involved in the study
☐	☐ Functional and/or effective connectivity
☐	☐ Graph analysis
☐	☐ Multivariate modeling or predictive analysis

Functional and/or effective connectivity

Graph analysis

Multivariate modeling and predictive analysis
