# Supplementary material for: A two-pronged strategy eliminates dissociation artifacts for high-fidelity neuroimmune single-cell transcriptomics
Source: Commun Biol. 2026 Jul 6;9:1294. doi: 10.1038/s42003-026-10609-x (PMC13642925; doi:10.1038/s42003-026-10609-x)
Supplement: Supplementary file 2 [file 42003_2026_10609_MOESM1_ESM.pdf]

A Two-Pronged Strategy Eliminates Dissociation Artifacts for High-Fidelity Neuroimmune Single-Cell Transcriptomics

Yuan Yan(鄢源)[1,2,3#], Bo Tang(唐博)[4#], Pu Liu(刘普)[2,3], Dongmei You(游东梅)[2,3], Yi Lv(吕铁)[4], Jinmeng Yi(义金蒙)[4], Yuzhang Wu(吴玉章)[2,4*] &Yiguo Qiu(邱一果)[2, 3*]

[1] School of Basic Medical Sciences, Southern Medical University, Guangzhou, China

[2] Chongqing International Institute for Immunology, Chongqing, China

[3] Sichuan-Chongqing Joint Key Laboratory of Traditional Chinese Medicine and Immunology, Chongqing, China

[4] Institute of Immunology, Third Military Medical University (Army Medical University), Chongqing, China

[#]These authors contributed equally.

*Corresponding authors:

Correspondence to qiu_yiguovivian@iiicq.vip and wuyuzhang@iiicq.vip

Supplementary Figures and Legends:

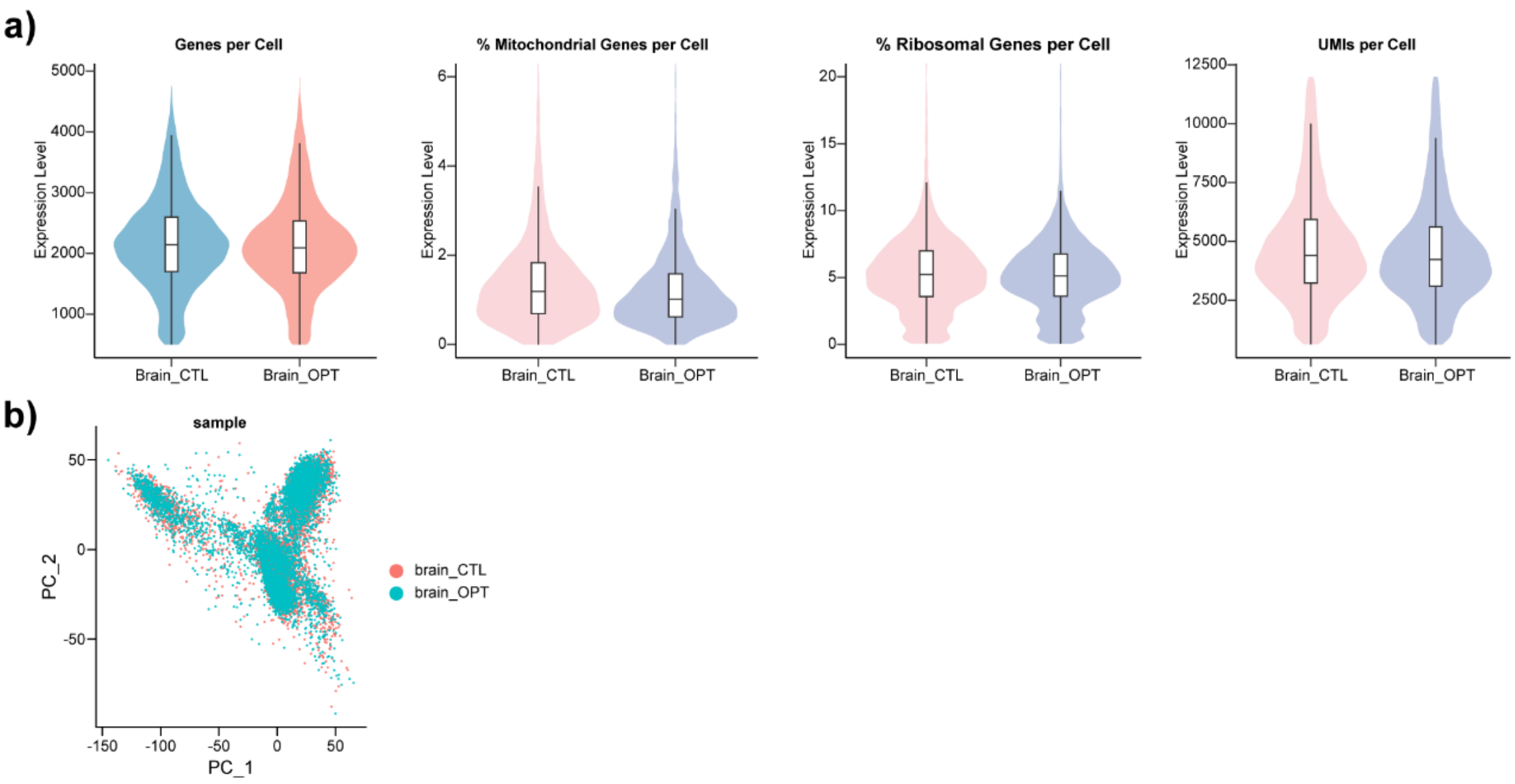

Supplementary Figure 1. Optimization of dissociation protocol preserves RNA quality and cell viability in brain tissue.

a, Quality control metrics for single-cell RNA sequencing of mouse brain cells processed with control (CTL) or optimized (OPT) dissociation protocols. Analysis of detected genes per cell, mitochondrial/ribosomal gene percentages, and UMI (Unique molecular identifier) counts per cell demonstrates comparable cell viability and RNA integrity between CTL and OPT conditions. Data shown for cells from 6 mice (n = 3 per group).

b, PCA plot of integrated single-cell datasets from brain_CTL and brain_OPT. Each point represents a single cell, colored by batch. Cells from different batches are well mixed in the PC1–PC2 space without clear separation, indicating minimal batch effects after integration.

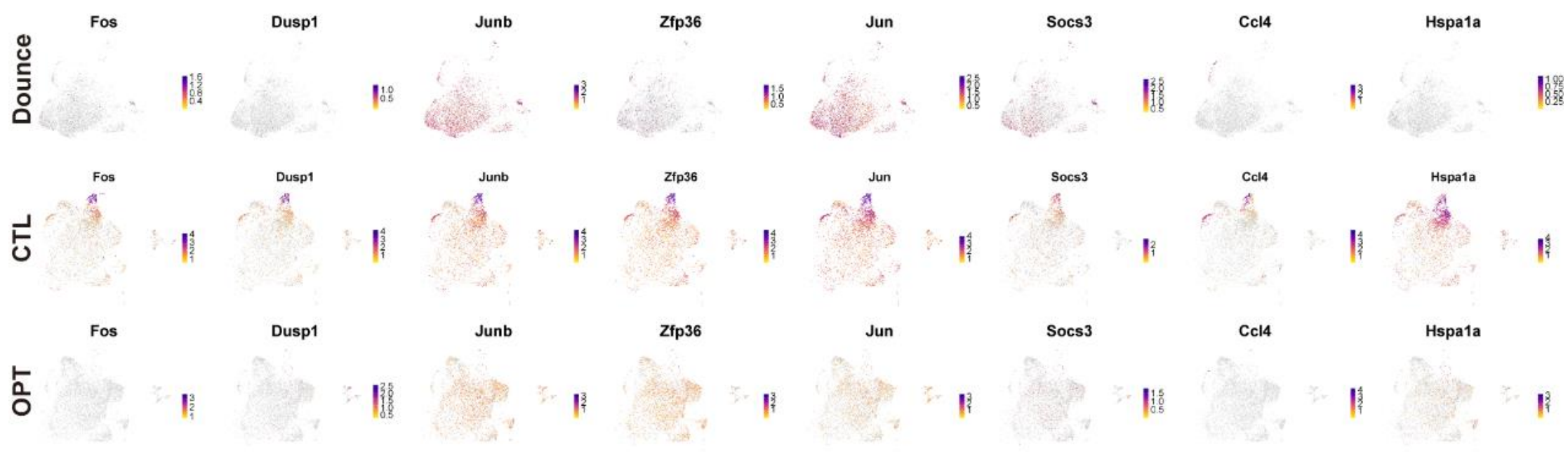

Supplementary Figure 2. Reduction of ex vivo activated microglia (exAM) signature genes in Dounce- and OPT-processed microglia.

Feature plots displaying the expression of representative exAM signature genes (e.g., *Fos, Dusp1, Junb, Jun, Socs3, Ccl4, Hspa1a* and *Zfp36*) in microglia from Dounce (top), CTL (middle), and OPT (bottom) groups.

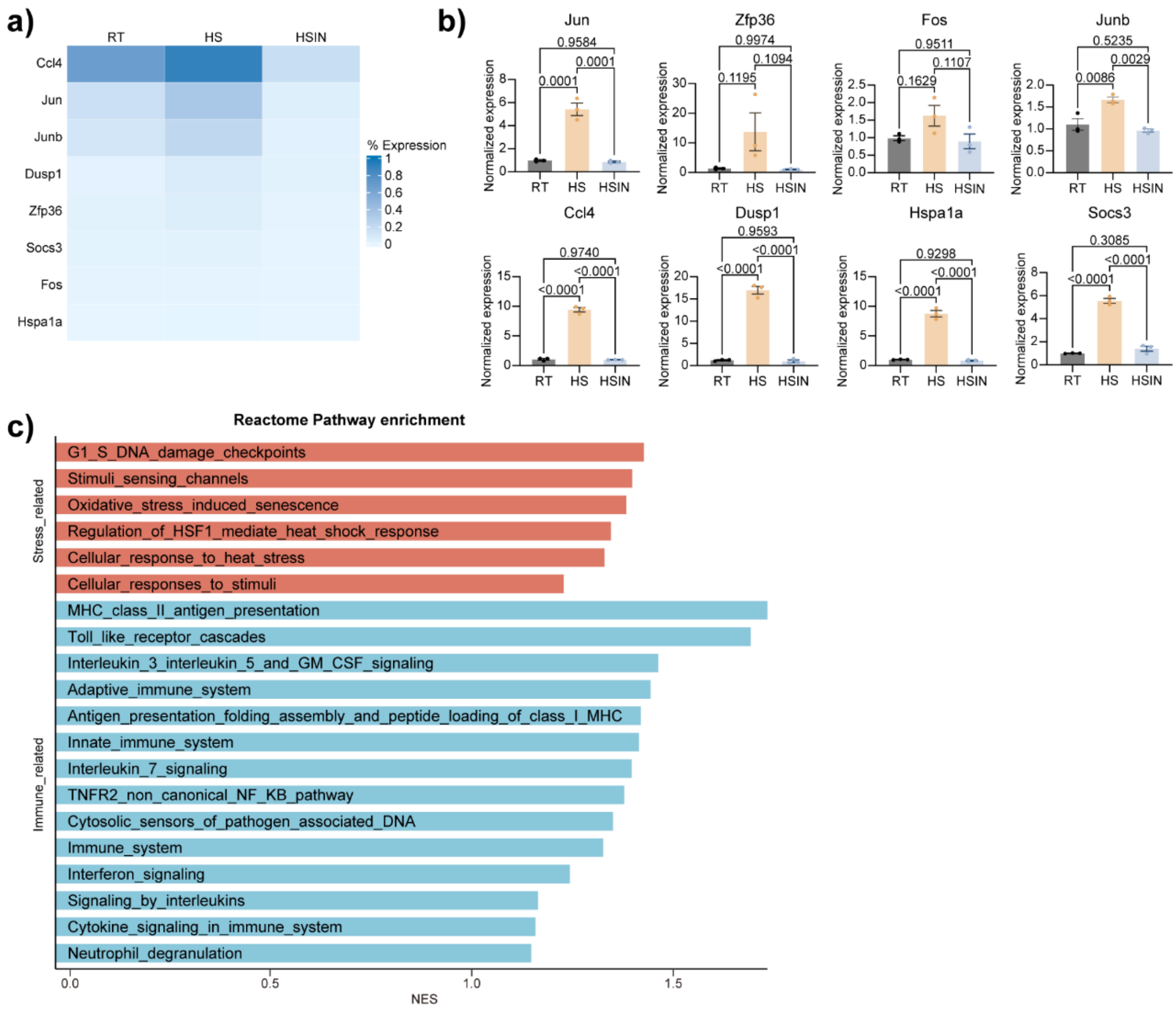

Supplementary Figure 3. In vitro characterization of stress-altered genes expression in BV2 microglia.

a, Heatmap showing expression levels for representative exAM signature genes in BV2

microglia from bulk RNA-seq under different conditions: room temperature (RT, 37 °C for 2 hours); heat stress (HS, 42 °C for 2 hours); heat stress with inhibitor cocktail (HSIN, 42 °C for 2 hours). Data shown for n=3 biological replicates.

b, Expression of representative exAM signature genes in BV2 microglia from RT-qPCR validation across three conditions: room temperature (RT, 37 °C for 2 hours); heat stress (HS, 42 °C for 2 hours); heat stress with inhibitor cocktail (HSIN, 42 °C for 2 hours). Relative expression was normalized by housekeeping gene *Gapdh* (n=3 biologically independent samples). Statistical significance was assessed with one-way ANOVA following by Tukey’s multiple comparisons test. Exact adjusted P values from Tukey's test are shown. The error bars indicate the Standard Error of the Mean.

c, Representative enriched pathways from Reactome database analysis of differentially expressed genes between RT and HS conditions in BV2 microglia. Among 71 significantly enriched pathways (Table S1, NES > 1, $p < 0.05$), 14 are immune-related (red) and 6 are stress-related (blue).

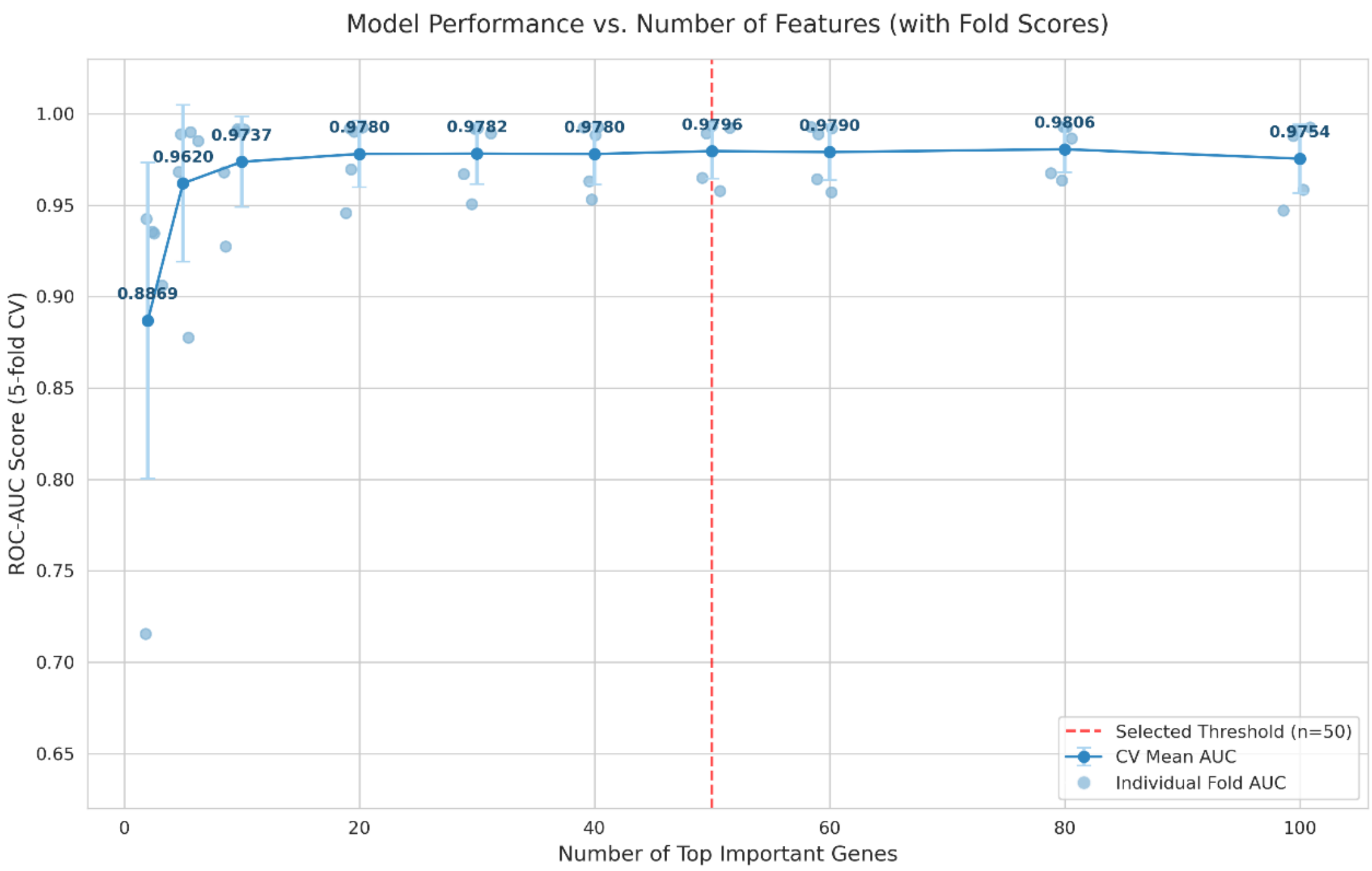

Supplementary Figure 4. Feature number selection based on model performance for brain.

Top-ranked genes were incrementally added as input features, and model performance was evaluated using 5-fold cross-validation (ROC-AUC). The performance gain diminishes beyond approximately 50 features, indicating limited benefit from additional features. Therefore, 50 was chosen as the optimal feature number (red dashed line). Each data point represents one independent validation (n=5 cross-validation in each feature size). The error bars indicate the standard deviation.

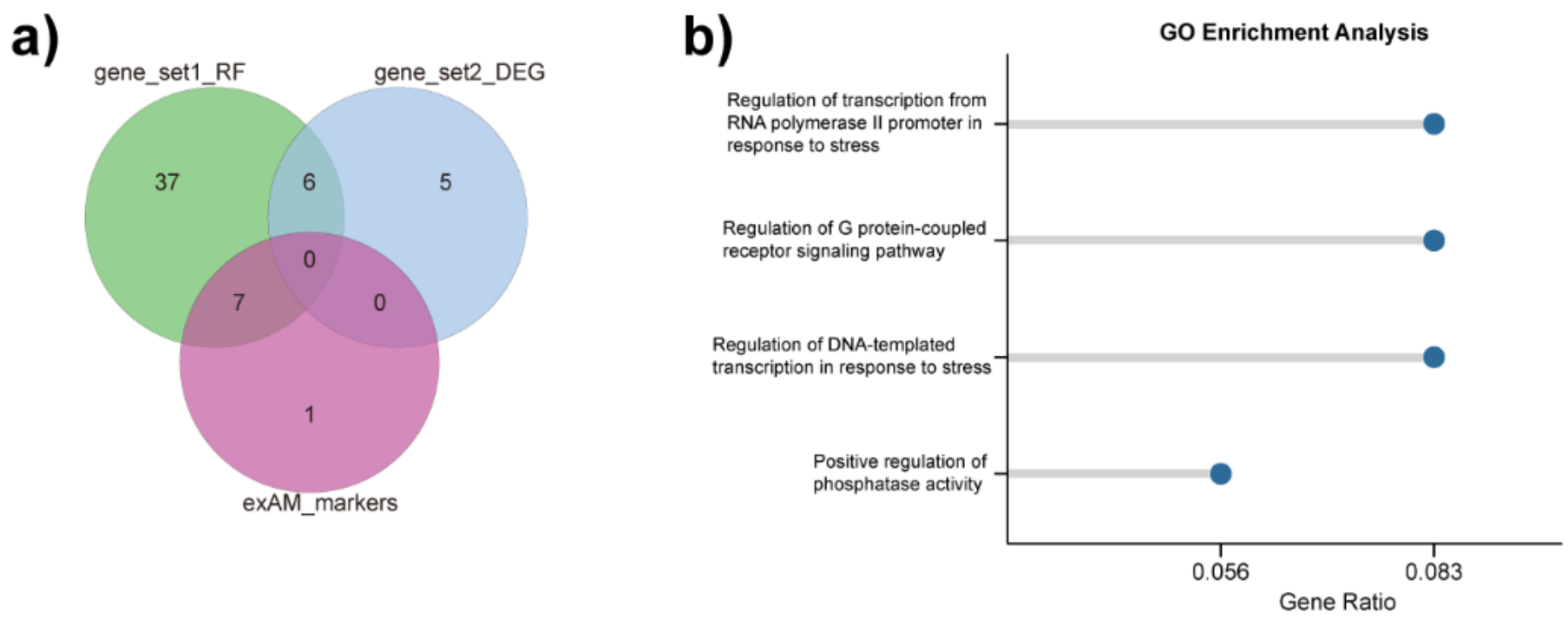

Supplementary Figure 5. Comparison of gene sets and functional enrichment analysis.

a, Venn diagram showing the overlap among gene_set1_RF, gene_set2_DEG, and exAM_markers. The gene_set1_RF exhibits limited overlap with the other gene sets and contains 37 unique genes.

b, Gene Ontology (GO) enrichment analysis of the 37 genes uniquely identified in gene_set1_RF. Representative enriched biological processes are shown. The x-axis indicates the gene ratio.

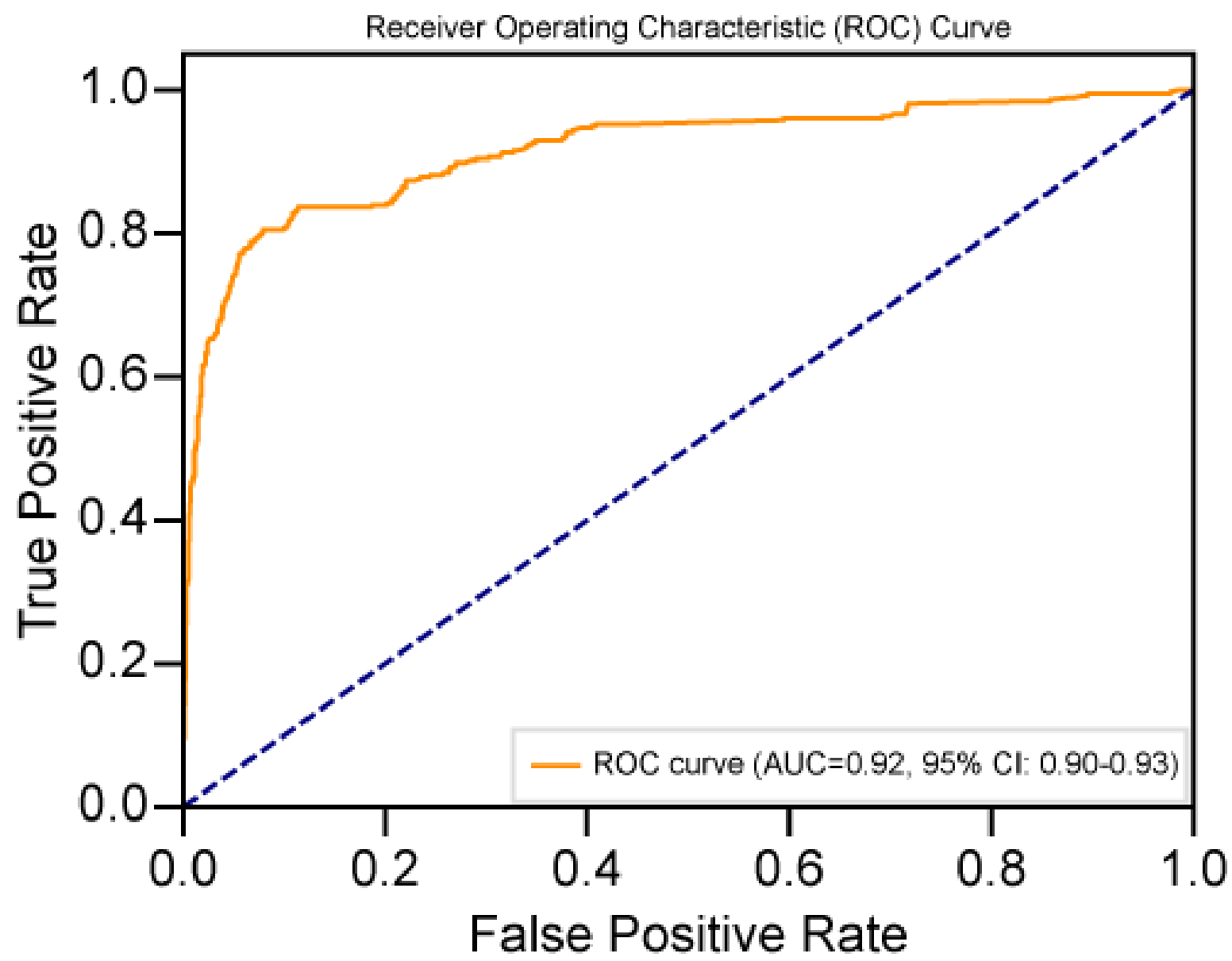

Supplementary Figure 6. External validation on independent biology replicates.

Receiver operating characteristic (ROC) curves evaluating brain-specific SAT signature performance in independent biological replicates. Model was trained for ex vivo activated microglia (GSE152182).

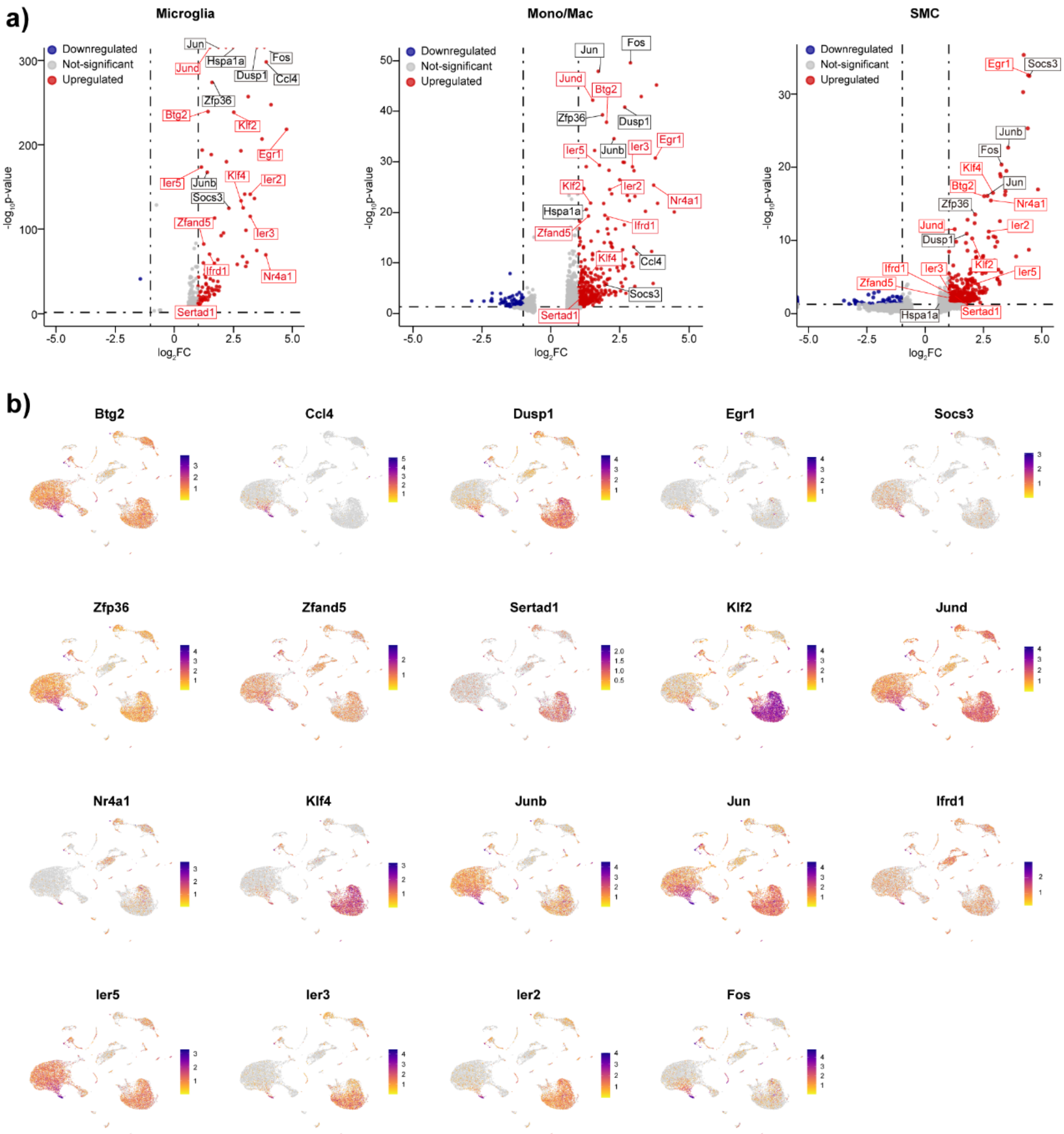

Supplementary Figure 7. Expression of representative brain-specific SAT genes.

a, Volcano plots of DEGs between exA-cells and homeostatic cells in microglia (left), monocytes/macrophages (middle), and SMCs (right). Significantly upregulated (red) and downregulated (blue) genes are highlighted. Black boxes indicate exAM signature genes; red boxes denote consistently upregulated SAT genes ($log_2FC > 1$).

b, Feature plots displaying 19 representative SAT genes across brain cells.

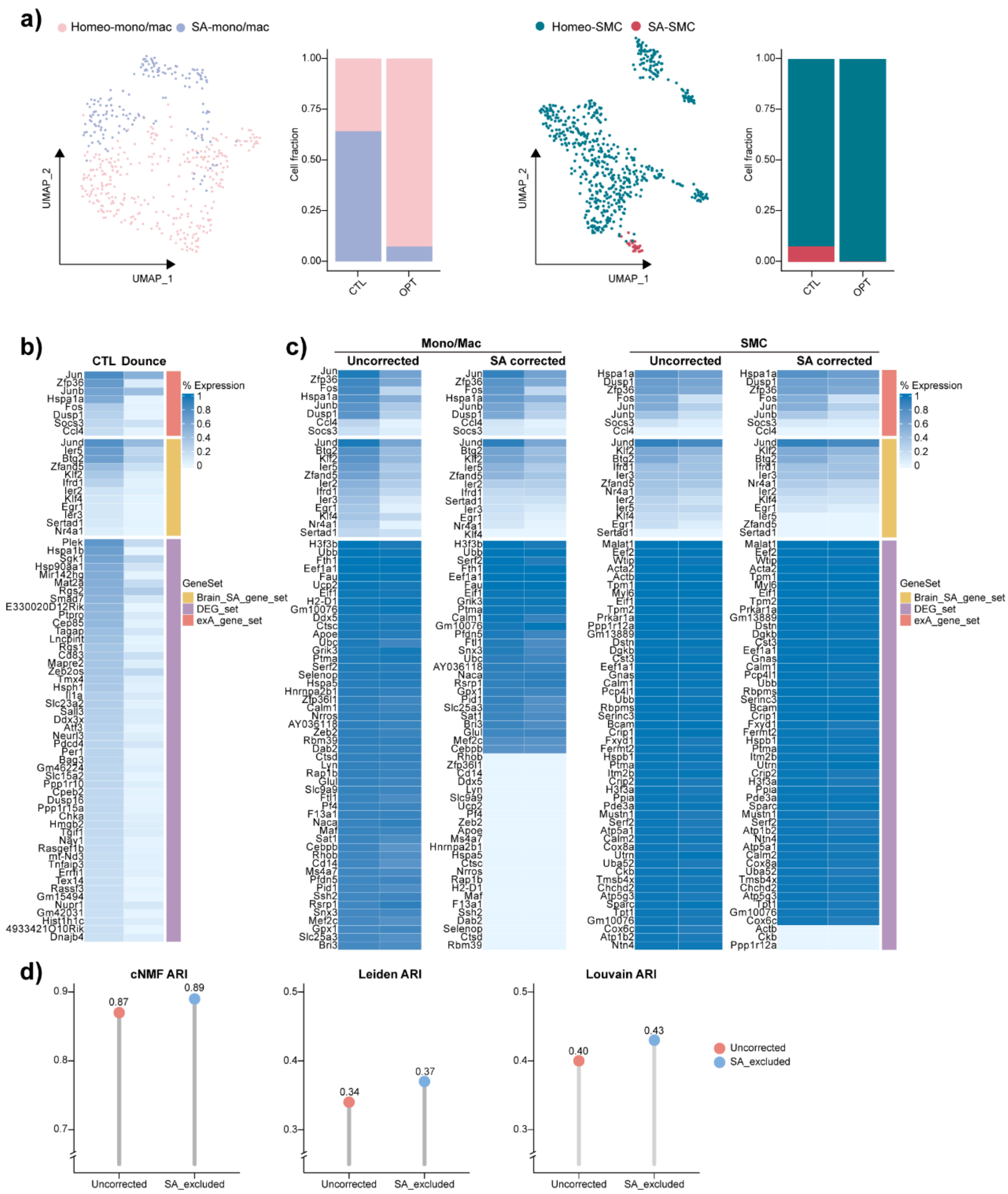

Supplementary Figure 8. SAT signature enables identification and computational correction of stress artifacts in brain cells.

a, Distribution of SAT signature-expressing cells in monocytes/macrophages (left) and SMCs (right), with bar graphs quantifying the proportion of SAT-positive cells.

b, Heatmap of top 50 DEGs between CTL and Dounce-isolated microglia, including SAT/exAM signature genes. Color bars denote expression levels and gene set classification.

c, Heatmaps comparing top 50 DEGs between CTL and OPT conditions in

monocytes/macrophages and SMCs before (left) and after (right) in silico removal of SAT-expressing cells. Color bars denote expression levels and gene set classification.

d, Adjusted Rand Index (ARI) comparisons between uncorrected and SA_excluded brain cells using cNMF (consensus non-negative matrix factorization, left), Leiden (middle) and Louvain (right) algorithms demonstrate improved clustering stability following computational removal of SAT-expressing cells.

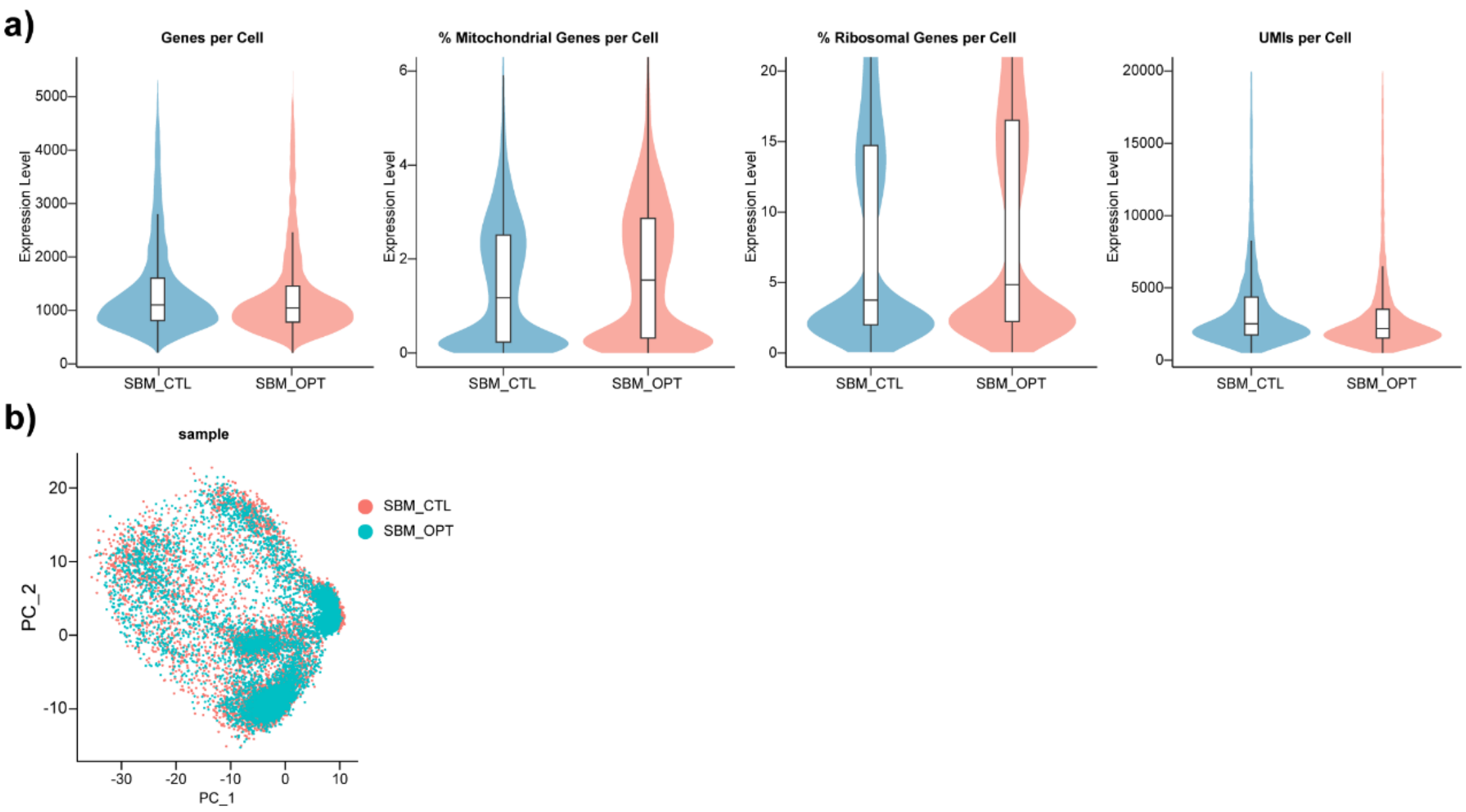

Supplementary Figure 9. Optimization of dissociation protocol preserves RNA quality and cell viability in SBM.

a, Quality control metrics for SBM cells processed with CTL or OPT dissociation protocols, showing comparable cell viability and RNA integrity based on gene detection, mitochondrial/ribosomal percentages, and UMI counts. Data shown for cells from 6 mice (n = 3 per group).

b, PCA plot of integrated single-cell datasets from SBM_CTL and SBM_OPT. Each point represents a single cell, colored by batch. Cells from different batches are well mixed in the PC1–PC2 space without clear separation, indicating minimal batch effects after integration.

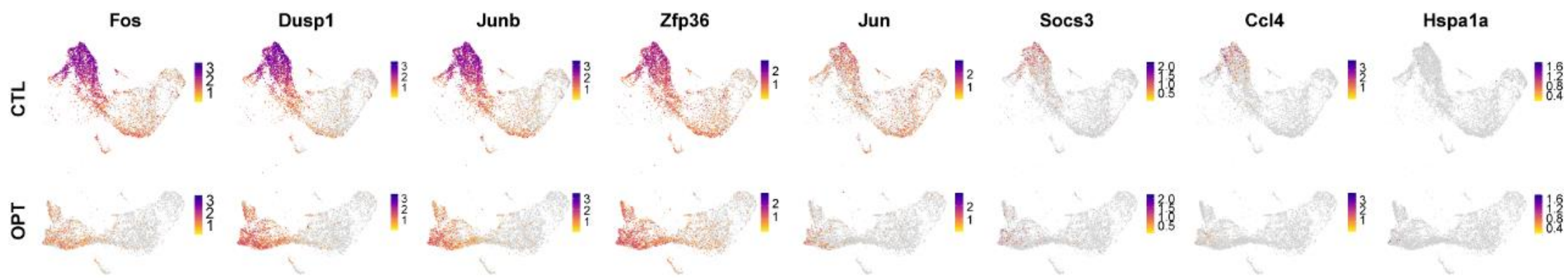

Supplementary Figure 10. Reduction of exA-gene signature genes in OPT-processed SBM neutrophils.

Feature plots displaying the expression of representative exA signature genes (e.g., *Fos, Dusp1, Junb, Jun, Socs3, Ccl4, Hspa1a* and *Zfp36*) in SBM neutrophils from CTL (top) and OPT (bottom) conditions.

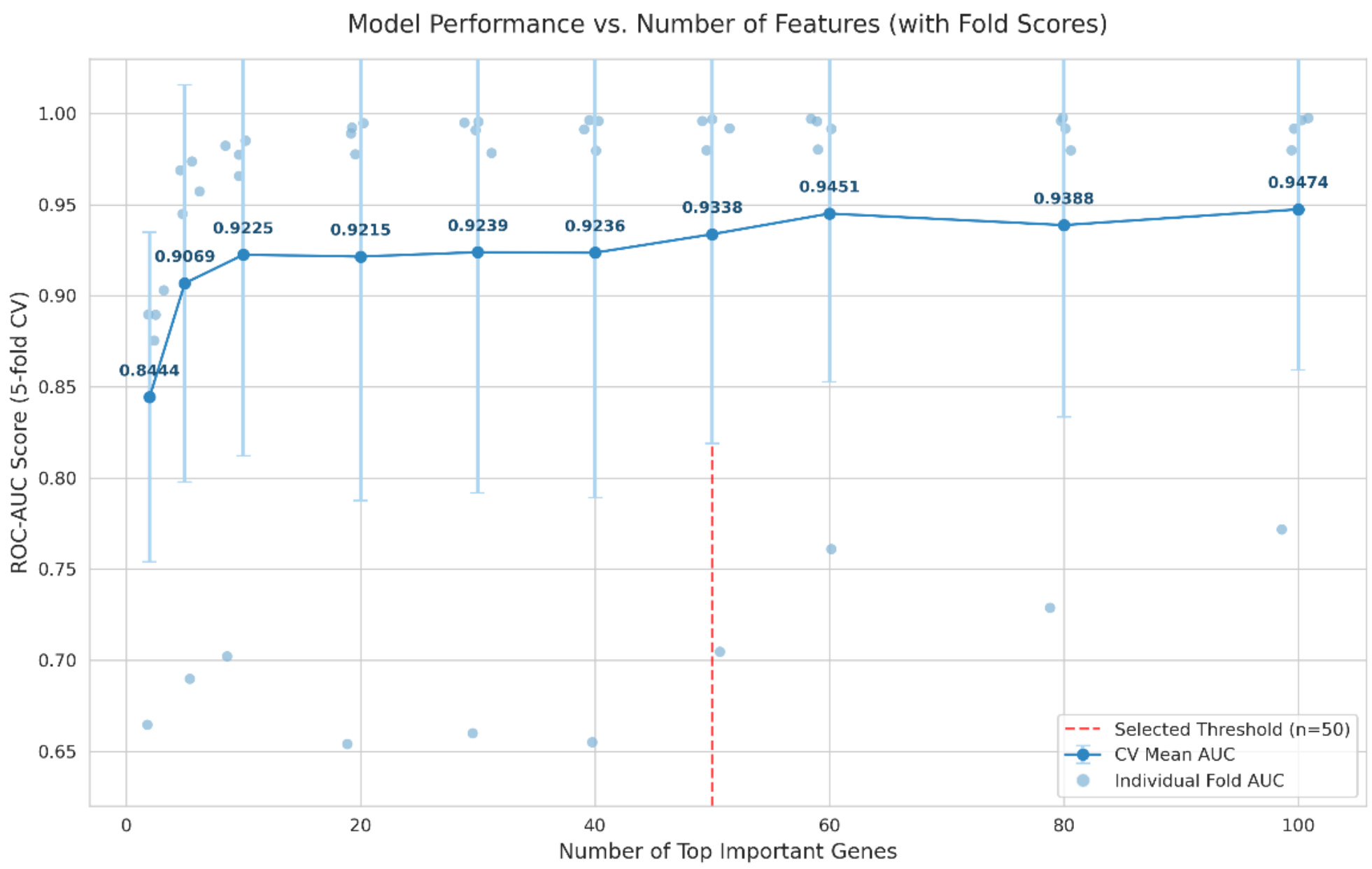

Supplementary Figure 11. Feature number selection based on model performance for SBM.

Top-ranked genes were incrementally added as input features, and model performance was evaluated using 5-fold cross-validation (ROC-AUC). 50 was chosen as the feature number (red dashed line). Each data point represents one independent validation (n=5 cross-validation in each feature size). The error bars indicate the Standard deviation.

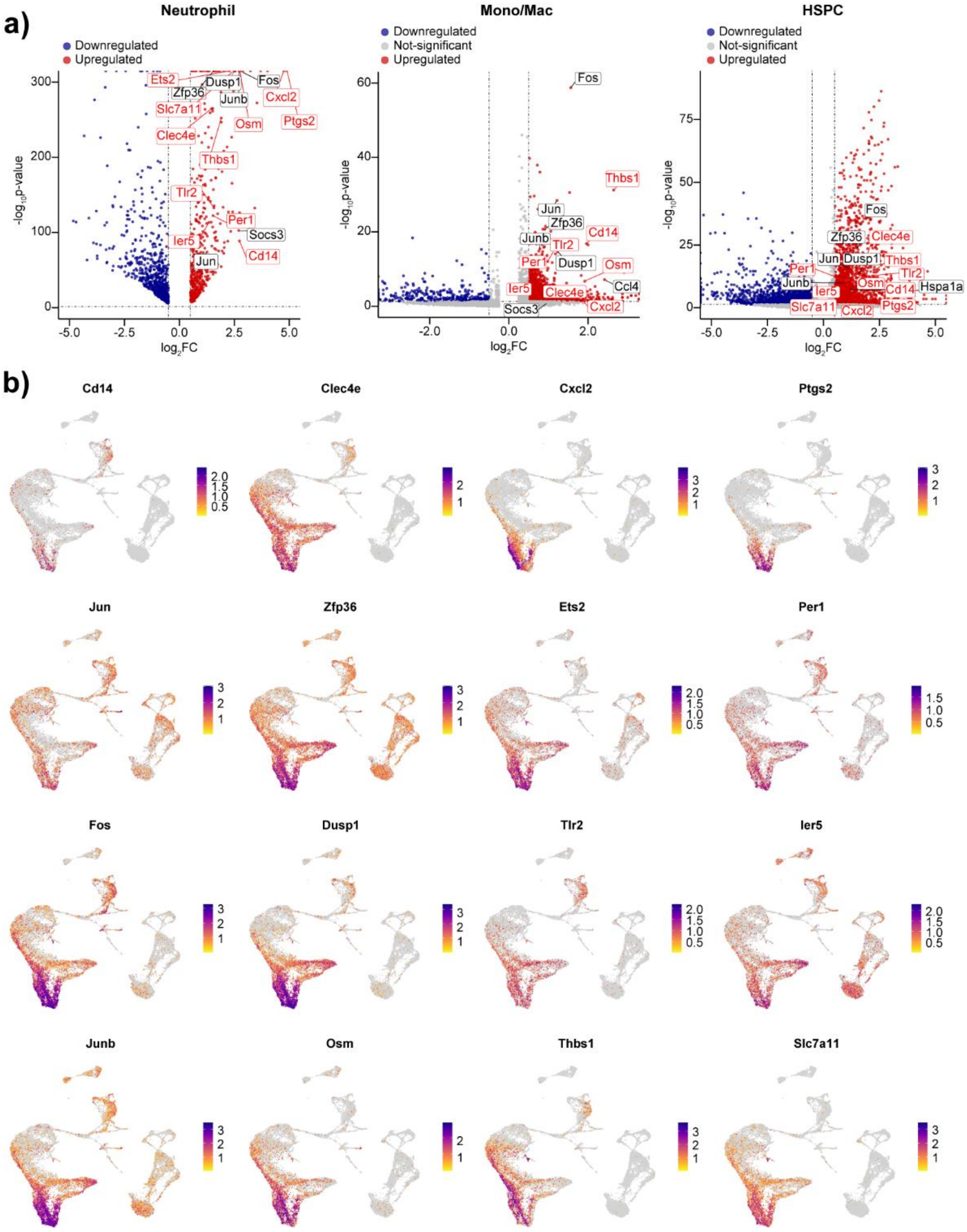

Supplementary Figure 12. Characterization of SBM-specific SAT signatures.

a, Volcano plots of DEGs between exA-cells and homeostatic cells in neutrophil (left), monocytes/macrophages (middle), and HSPCs (right). Significantly upregulated (red) and downregulated (blue) genes are highlighted with exA (black boxes), and SAT (red boxes) signatures indicated ($log_2FC > 1$).

b, Feature plots displaying 16 representative SAT genes across SBM cell populations.

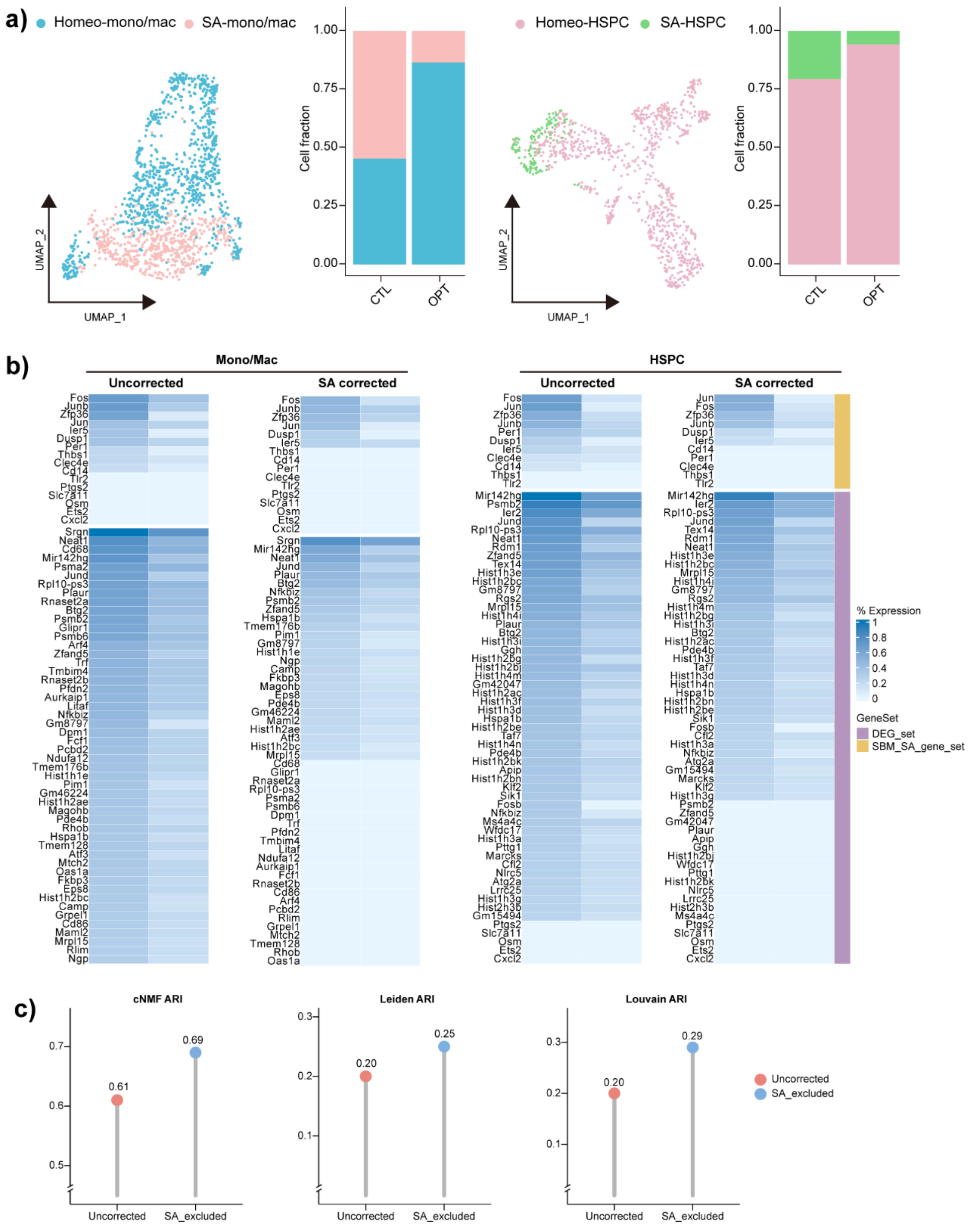

Supplementary Figure 13. SAT signature enables identification and computational correction of stress artifacts in SBM cells.

a, UMAP plots displaying the distribution of SAT signature-expressing cells in monocytes/macrophages (left), and HSCPs (right), with bar graphs quantifying SAT-positive cell proportions.

b, Heatmaps comparing top 50 DEGs and the SAT/exA signature genes between CTL and OPT conditions in monocytes/macrophages and HSPCs before (left) and after (right)

computational removal of SAT-expressing cells. Color bars denote expression levels and gene set classification.

c, Adjusted Rand Index comparisons using cNMF (left), Leiden (middle) and Louvain(right) algorithms demonstrate improved clustering stability in SBM following computational removal of SAT-expressing cells.

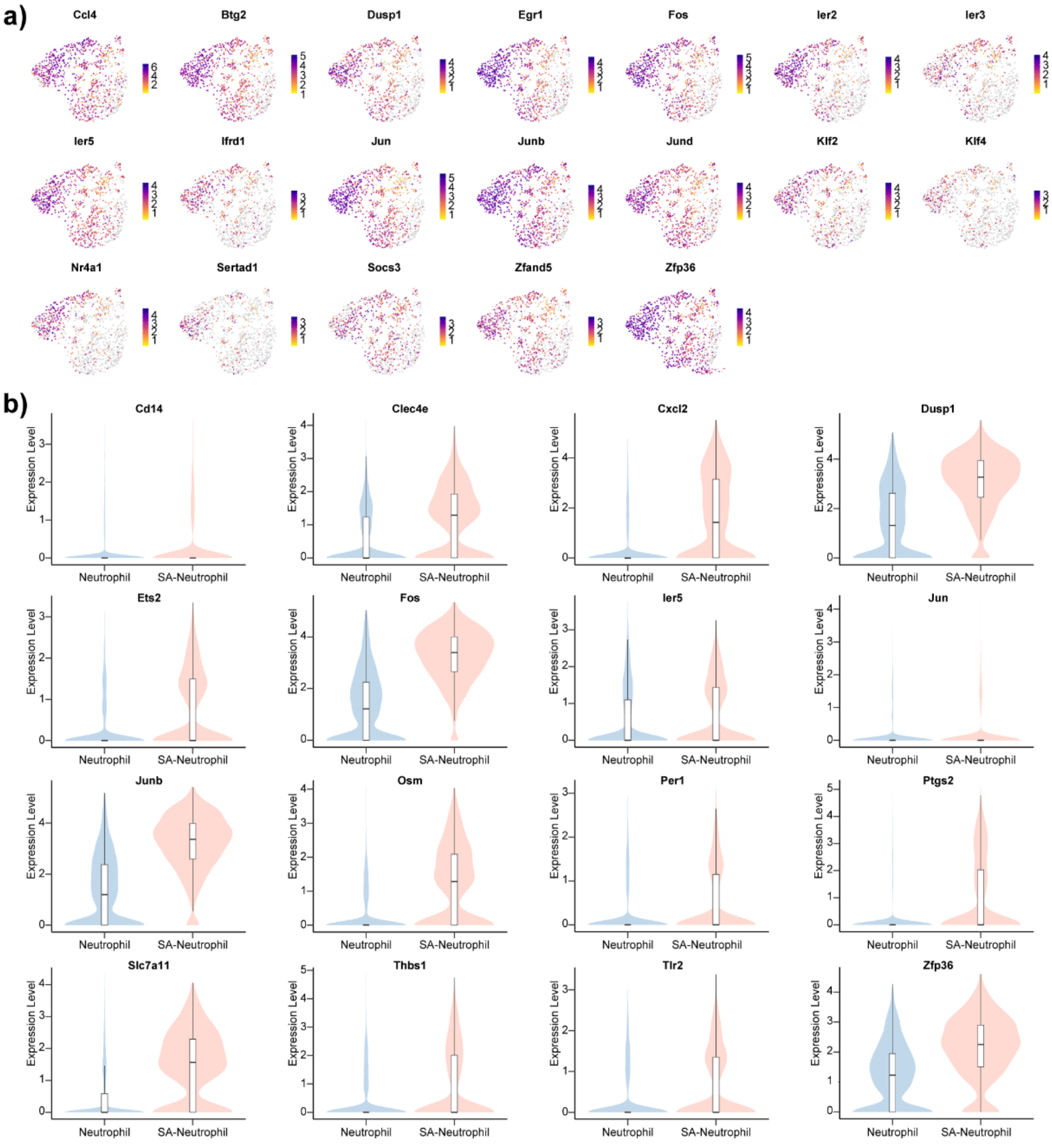

Supplementary Figure 14. Validation of SAT signatures in published datasets.

a, Feature plots displaying 19 brain-specific representative SAT genes in microglial data from Wang et al. (2025).

b, Comparison of 16 SBM-specific representative SAT genes in SBM data from Kolabas et al. (2023).

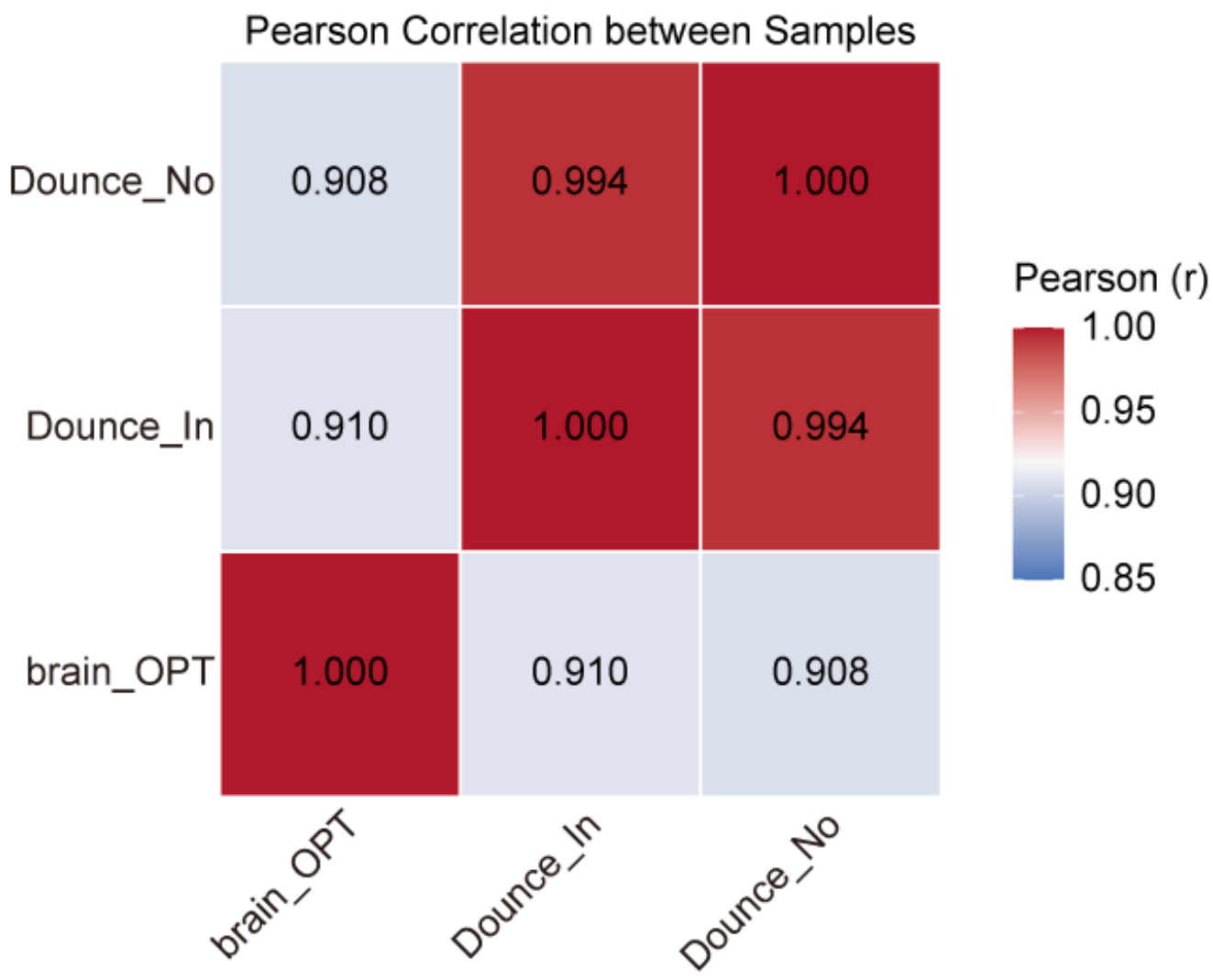

Supplementary Figure 15. Spearman correlation analysis of transcriptomic profiles

Complete transcriptome profiles for microglia that collected from Brain_OPT, Dounce_in (with inhibitor, GSE152183) and Dounce_No (without inhibitor) are used for correlation analysis.
