# Supplementary material for: A two-pronged strategy eliminates dissociation artifacts for high-fidelity neuroimmune single-cell transcriptomics
Source: Commun Biol. 2026 Jul 6;9:1294. doi: 10.1038/s42003-026-10609-x (PMC13642925; doi:10.1038/s42003-026-10609-x)
Supplement: Supplementary file 3 [file 42003_2026_10609_MOESM2_ESM.pdf]

Description of Additional Supplementary Files

File name: Supplementary Data 1

Description: 71 Reactome pathways for BV2 (RT vs HS

File Name: Supplementary Data 2

Description: Genes importance ranking list for Brain

File Name: Supplementary Data 3

Description: 53 SAT signature gene list for brain

File Name: Supplementary Data 4

Description: Geneset1(RF)_geneset2(findmarker)_exAM markers

File Name: Supplementary Data 5

Description: Pathway enrichment of geneset1(RF) unique genes

File Name: Supplementary Data 6

Description: Genes importance ranking list for SBM

File Name: Supplementary Data 7

Description: SAT signature gene list for SBM

File Name: Supplementary Data 8

Description: GSEA enrichment for brain and SBM

File Name: Supplementary Data 9

Description: Class-balance matrix for model testing and validation

File Name: Supplementary Data 10

Description: Primer sequence for qPCR

File Name: Supplementary Data 11

Description: Numerical Source data for graphs
